# Chitosan Hydrogels Cross-Linked with Trimesic Acid for the Delivery of 5-Fluorouracil in Cancer Therapy

**DOI:** 10.3390/pharmaceutics15041084

**Published:** 2023-03-28

**Authors:** Sravani Emani, Anil Vangala, Federico Buonocore, Niousha Yarandi, Gianpiero Calabrese

**Affiliations:** School of Life Sciences, Pharmacy and Chemistry, Kingston University London, Kingston-upon-Thames KTI 2EE, UK

**Keywords:** chitosan, 1,3,5-benzene tricarboxylic acid, hydrogels, rheology

## Abstract

Chitosan exhibits unique properties making it a suitable material for drug delivery. Considering the rising popularity of hydrogels in this field, this work offers a comprehensive study of hydrogels constituted by chitosan and cross-linked with 1,3,5-benzene tricarboxylic acid (BTC; also known as trimesic acid). Hydrogels were prepared by cross-linking chitosan with BTC in different concentrations. The nature of the gels was studied through oscillatory amplitude strain and frequency sweep tests within the linear viscoelastic region (LVE) limit. The flow curves of the gels revealed shear thinning behavior. High G′ values imply strong cross-linking with improved stability. The rheological tests revealed that the strength of the hydrogel network increased with the cross-linking degree. Hardness, cohesiveness, adhesiveness, compressibility, and elasticity of the gels were determined using a texture analyzer. The scanning electron microscopy (SEM) data of the cross-linked hydrogels showed distinctive pores with a pore size increasing according to increasing concentrations (pore size range between 3–18 µm). Computational analysis was performed by docking simulations between chitosan and BTC. Drug release studies employing 5-fluorouracil (5-FU) yielded a more sustained release profile with 35 to 50% release among the formulations studied in a 3 h period. Overall, this work demonstrated that the presence of BTC as cross-linker leads to satisfactory mechanical properties of the chitosan hydrogel, suggesting potential applications in the sustained release of cancer therapeutics.

## 1. Introduction

Polymeric hydrogels are used in the field of drug delivery for the controlled release of therapeutic ingredients. Hydrogels are cross-linked networks and hold hydrophilic functional groups in their structure. Hydrophilic functional groups, such as amine (-NH_2_)_,_ hydroxyl (-OH), sulfonates (-SO_3_H), and amides (CONH_2_), can absorb large amounts of water and are attached to polymeric networks [1]. They form a mesh-like structure that has a potential ability to hold and deliver drug molecules at the site of action [2,3]. The hydrogels can be formed from either natural (chitosan [4], collagen [5], fibrin [6], cellulose [7], hyaluronic acid [8]), synthetic (polyethylene glycol [9], polyvinyl alcohol [10], polyacrylamide [11], and poly-N-isopropylacrylamide [12]) or semisynthetic materials (PEG with other proteins) [13]. The polymeric chains establish a superficial 3D network with interstitial spaces that can port the physiological or aqueous fluids [14]. These fluids can facilitate the diffusion of oxygen as well as nutrients that play an important role in cell growth and proliferation [15].

**Figure 1:**
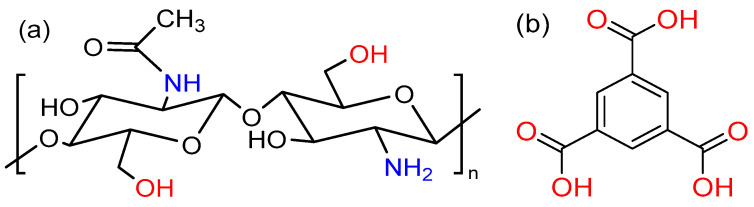
Structures of (**a**) chitosan and (**b**) 1,3,5-benzene tricarboxylic acid (BTC).

Among several polymers, chitosan is extensively studied in drug delivery. A great number of applications have been proposed for chitosan, from HIV [16] and cancer therapy [17,18,19,20,21,22], to tissue engineering [23], biotechnology, agriculture [24], and wound dressings [25,26,27,28]. Chitosan is a cationic polymer made of repeated units of *β*-(1 → 4)-2-acetamido-D-glucose and *β*-(1 → 4)-2-amino-D-glucose units and formed via the process of deacetylation of chitin. The chemical structure of chitosan is composed of two main sugars, namely glucosamine and N-acetyl glucosamine (Figure 1). The chemical reactivity of the polymer is due to the presence of primary amino and hydroxy groups. 

At physiological pH, chitosan acts as a mucoadhesive polymer. Here, the amino groups become protonated and act as a determining factor in chitosan’s mucoadhesive properties [29]. When dissolved in protic solvents, such as water, chitosan possesses positively charged amine groups (NH_3_^+^), which account for its solubility [25] and its mucoadhesive properties (as it can favorably interact with the negatively charged mucus). The degree of deacetylation (DD) and the molecular weight of chitosan are also crucial in determining biological properties. The solubility of the polymer increases with an increase in DD of chitosan, but on the other hand, a low DD results in a slower diffusion of the drug through the polymeric network. Hence, chitosan with an approximate DD value of 75–85% is preferred [30]. 

Chitosan is in the form of either physical or chemically cross-linked hydrogels. Cross-linkers interact with the amine groups in the chitosan, forming molecular bridges in the chitosan chains. As novel chitosan and carrageenan nanoparticles-based gels showed a good potential for the sustained release of drugs in topical administration [31], more recently, research focused on the hydrogels produced by cross-linking chitosan with different materials, such as glutaraldehyde [32,33,34], genipin [35,36], and collagen [37,38]. One such material used for this purpose is 1,3,5-benzene tricarboxylic acid (BTC, Figure 1). Controlled drug release of the chitosan–BTC hydrogels have previously been reported by Yang et al. [39]. In addition, the positively charged chitosan in acidic medium can allow the attachment of nucleic acids, such as DNA and siRNA [40]. The reason for using BTC is to design a more stable system that can encapsulate both hydrophilic and lipophilic drugs in the polymeric networks [41]. Hydrogels with definite porous structures can be used as carriers for drug delivery [42]. The carboxylic acid (-COOH) groups of BTC undergo deprotonation and form ionic cross-links with the amine groups (NH_2_) of chitosan. In addition, possible interactions, such as ionic, hydrogen, and π-π bonding, were reported in the literature [43]. 

When combining chitosan and BTC, gelation occurs at room temperature, and these gels can be used for encapsulating hydrophilic and lipophilic drugs for controlled or sustained drug release. In previously reported procedures, porous chitosan was prepared using BTC as a cross-linker [39] and supramolecular hydrogels from hydroxy pyridines [44]. In biomedical applications, there is a need for a stable hydrogel system that meets essential criteria of toughness and stability. However, most hydrogels formulated do not display these properties. Hence, there is a need to improve the gel characteristics either by physical or chemical alteration. The rheological studies of the viscoelastic materials provide the information about the mechanical strength of the hydrogels [45]. The dynamic changes in the microscopic structure and gelation can be monitored using a rheometer [46]. This aids in understanding the cross-linking degree, elasticity, flow, and viscosity of the gel material in response to applied stress and strain [47]. The rheological tests can be performed monitoring parameters, such as stress relaxation (when subjected to strain, there is decrease in stress), oscillatory amplitude strain sweep (amplitude of the deformation or shear stress is varied at constant frequency), frequency strain sweep within the linear viscoelastic region (LVE), rotational viscometry, and creep recovery [48]. 

**Figure 2:**
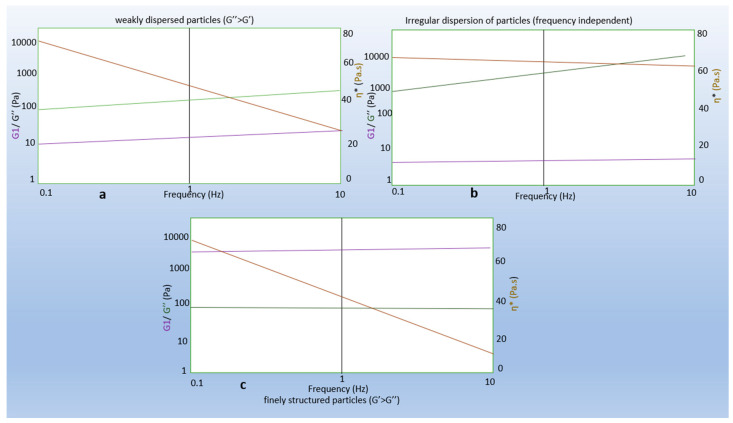
Graphs representing viscoelastic parameters: (**a**) weakly dispersed particles (G″ > G′); (**b**) irregular dispersion of particles; (**c**) fine structured particles (G′ > G″) [49]. Lines legend: purple for G′, elastic modulus; green for G″, viscous modulus; and orange for η*, complex viscosity...

Viscoelastic materials exhibit a reversible response that depends on the rate of applied load. This mechanism usually occurs in polymeric materials. These materials are time-dependent, which makes them strain-rate sensitive. Viscoelasticity is the property of a material that exhibits both viscous and elastic properties when deformed. When a viscoelastic material is subjected to stress, the response is composed of elastic deformation (which stores energy) and viscous flow (which releases energy). The profile of the frequency sweep data gives the degree of dispersion, whereas interparticle association frequency sweep curves can provide information about the product (gel) behavior during storage and application. At certain frequency, either the elastic or viscous seems dominant which indicates the elastic or viscous nature of the structured material (Figure 2). From Graph a (Figure 2), the particles were dispersed irregularly, and the viscous component dominated the elastic component [49]. In Graph b of the same figure, the loss modulus is greater than the viscous modulus, and complex viscosity (η*) is dependent on frequency. In both cases, sedimentation is likely to occur, causing the instability of the gel structure. In Graph c (Figure 2), the particles are evenly spread and well-dispersed. The elastic modulus dominated the viscous modulus and independence of frequency. 

The phase angle reveals the material deformation to either solid or liquid. Low phase angle results can be seen in a solid, and a high phase angle corresponds to liquid. The gel point is another critical parameter that determines the dynamic viscoelastic properties of the polymer systems. The sudden change in viscosity of the fluid can be determined using the gelling point. At the gelling point, a solution becomes more resistant to flow due to a loss in fluidity. Winter and Chambon [50,51] explained that the gelling point can be identified from the dynamic viscoelastic parameters. They emphasized the rheological parameters of a new cross-linking system using end-linked poly dimethyl siloxane. The viscoelasticity of the material can be determined from the loss tangent (tan δ) which is a ratio of the energy lost to the total energy stored during the analysis [52]. These measurements are important to determine the mechanical strength of the material. Creep recovery can provide information about the recoverable viscoelastic deformation and viscous deformation (non-recoverable) [53]. 

Drugs are released from the polymeric network through four different mechanisms: stimulated release, degradation-controlled, solvent-controlled, and diffusion-controlled release [54]. Thus, the rate of drug release depends on the characteristics of the polymer, solvent used, and the physico–chemical properties of the drug. Researchers have found that 5-FU has a promising anticancer effect and can be used as a model drug [55]. The efficacy and safety of 5-FU can theoretically be improved by using drug delivery vehicles such as hydrogels. Indeed, the high water content of the porous three-dimensional structure of chitosan hydrogels (up to 99% *w*/*w* of water in some cases) facilitates the incorporation of hydrophilic drugs. The non-specific distribution of the drug in vivo, which affects non-target cells leading to side effects, can be overcome by the introduction of hydrogels to release drugs in a controlled manner [56,57]. In this study, hydrogels were prepared by mixing physical solutions of chitosan and BTC. The prepared hydrogels were characterized using NMR, Fourier-transformed infrared spectroscopy (FTIR), texture analysis, and rheological data to determine the mechanical and flow properties of the hydrogels. The release of 5-FU from the prepared hydrogels and the impact of variations in cross-linker amounts were also investigated.

## 2. Materials and Methods

### 2.1. Materials

Low molecular weight chitosan (>75% deacetylated, 20–300 cps (1% in 1% acetic acid)), 1,3,5-benzene tricarboxylic acid (BTC, trimesic acid, C_9_H_6_O_6,_ Mwt 210.14 g/mol), 5-fluorouracil (5-FU, ≥99% HPLC, powder, C_4_H_3_FN_2_O_2,_ Mwt 130.08), dialysis tubing with an average flat width of 25 mm (1.0 inch) and molecular weight cut-off (MWCO) 3500 Da, glacial acetic acid, deuterium chloride, deuterium oxide, and ethanol were all purchased from Sigma-Aldrich, Gillingham (UK). Aluminium specimen stubs (0.5″) and carbon tabs (12 mm diameter) were purchased from Agar Scientific Ltd., Stanstead (UK). Deionised water was used for all preparations.

### 2.2. Preparation of Chitosan–BTC Hydrogels

Chitosan hydrogels with different concentrations were prepared according to a modification of the method previously reported by Yang et al. [39]. Briefly, chitosan solution (1 wt%, 2.50 g) was prepared by dissolving chitosan in acetic acid (250 mL of 0.6% *v*/*v*). This mixture was heated at 40 °C overnight. BTC solutions were prepared by dissolving BTC powder in ethanol in varying concentrations. Three different concentrations (M1, M2, M3) were, respectively, prepared by mixing:

- A combination of 1 wt% chitosan solution and 10 mM BTC-ethanol solution (M1);
- A combination of 1 wt% chitosan solution and 50 mM BTC-ethanol solution (M2);
- A combination of 0.5 wt% chitosan solution and 50 mM BTC-ethanol solution (M3).

The solutions of chitosan and BTC formed a gel at room temperature upon mixing. 

A consistent amount of drug (15 mg) was loaded in all formulations to have a final concentration of 0.3 mg/mL of 5-FU.

### 2.3. Computational Study

The docking procedure for subsequent ligands was performed using AutoDock Vina (docking software) [58] and UCSF Chimera (software to display the docking) [59]. The SMILES strings for the molecules were obtained using SwissADME [60].

### 2.4. Nuclear Magnetic Resonance (NMR) Spectroscopy

The typical proton NMR spectra of chitosan and chitosan–BTC were performed on a Bruker Avance III 400 MHz two-channel FT-NMR spectrometer. An aliquot of lyophilized sample (c.a. 15 mg) was dissolved in DCl/D_2_O (20%). After dissolution, the chitosan solution was transferred to an NMR tube (5 mm). The experiments were carried out at 70 °C, a temperature at which solvent peaks does not interfere with the chitosan’s peaks. The chemical shifts of the chitosan and BTC protons were recorded using the spectra. 

### 2.5. Fourier Transform INFRARED Spectroscopy (FTIR)

Hydrogel formulations were analyzed using a thermo-scientific Nicolet^TM^ iS5 FTIR spectrophotometer in the range of 4000–400 cm^−1^ with an accumulation of 16 scans. The sample spectrum was collected and processed using OMNIC thermo scientific software.

### 2.6. Viscosity

The viscosity of the hydrogels was determined at room temperature using a Brookfield dial (DV-II + Pro) viscometer. The formulations were placed in beakers (25 mL), and measurements were taken using an LV spindle 64. The viscosity readings (in cP) were recorded at different shear rates (10, 20, 50, 60, 100 rpm) with the torque range between 10 and 100%. The viscometry parameters were measured at 25 ± 0.05 °C in triplicates. 

### 2.7. Texture Profile Analysis 

The texture profile analysis (TPA) of the hydrogels was carried out using a TA.XT.Plus texture analyser (Stable Micro System). This instrument can be used to determine the physical characteristics, cohesiveness, adhesiveness, consistency, and firmness of gels or other semisolid dosage forms [61,62]. The test results can be correlated to the therapeutic outcome of the drug formulation [63]. In addition, this is a simple and reproducible method that provides an easy method to perform the testing of the gels. The probe used for the analysis of the formulations was P/10. The probe was programmed at the selected speed of 2 mm/s, pre-test speed (1.0 mm/s), and post-test speed (10 mm/s) at 5 mm. The instrument was calibrated (including the probe height) before the sample was tested. Approximately, the gel formulation (25 mL) was placed in a standard beaker (50 mL), and care was taken to avoid air bubbles that can interfere with the results. The readings were taken in triplicates.

### 2.8. Rheological Characterization

Rheological measurements of the chitosan–BTC hydrogels were carried out using a rotational Malvern Kinexus Pro rheometer using rSpace software. The hydrogels were tested after 7 days of preparation using the probe CP4/40 SR4147SS. Measurements were taken at 37 °C for all samples. The samples were dispensed on the surface of the preheated lower plate, and the upper cone was allowed a gap of 0.1424 mm. The excess hydrogel was removed using a spatula. Then, the temperature was allowed to equilibrate to 37 °C. Once stabilized, the flow curves were recorded with varied shear rates at the same temperature. The tests were performed in triplicates.

Oscillation amplitude sweep and frequency sweep tests within the linear viscoelastic region (LVE) were carried out at 37 °C. Strain sweep tests were performed using a strain from 0.1 to 100% to the hydrogel once the gel was allowed to reach equilibrium. These tests measure the storage and loss modulus with respect to shear strain at constant temperature (37 °C) and frequency (1 Hz). In the frequency sweep, the frequency was varied (0.1 to 10%), while the amplitude of the deformation was kept constant. The storage and loss modulus of the hydrogels were plotted against frequency to determine the viscoelastic properties of the gels. 

### 2.9. Scanning Electron Microscopy

The morphology of the hydrogels was analysed using a Zeiss EVO 50 scanning electron microscope (SEM). The hydrogels were freeze-dried and positioned on aluminum specimen stubs on which 12 mm diameter carbon tabs were placed. The gels were coated with gold using a sputter coater (SC7640) and scanned at an extra high-tension voltage of 10–20 kV. The specimen was adjusted to a height of 2 mm with a diameter of 35 mm. The samples were viewed using different magnifications, and the software used for SEM operation was Zeiss Smart SEM.

### 2.10. Drug Release Study

The release of 5-FU was studied at 37 °C. Briefly, samples (5 mL of hydrogel, concentration of 5-FU = 0.3 mg/mL) were placed in a dialysis bag and kept in phosphate buffered saline (PBS solution) (50 mL) at pH 7.4 [64]. At regular intervals, 2 mL of the release medium was removed from the solution and replaced with fresh medium (2 mL). The drug release study was monitored up to 180 min. The same method was employed for studying the release of 5-FU at pH = 6.5. UV-Vis spectrophotometry at 266 nm was used to determine the amount of 5-FU released. The total amount of 5-FU loaded and the cumulative release were calculated from different concentrations of drug solutions using a standard calibration curve at 266 nm (R^2^ = 0.9912). 

### 2.11. Statistical Analysis

Statistical differences in the drug release profile were assessed using an ANOVA test [65], and a value of *p* < 0.05 was considered statistically significant. The results from the experimental data were presented as mean ± standard deviation. The error bars in the graph represent the standard deviation (n = 3). The results attained were analyzed using SPSS^®^ statistical software.

## 3. Results and Discussion

### 3.1. Interaction between Chitosan and BTC

**Figure 3:**
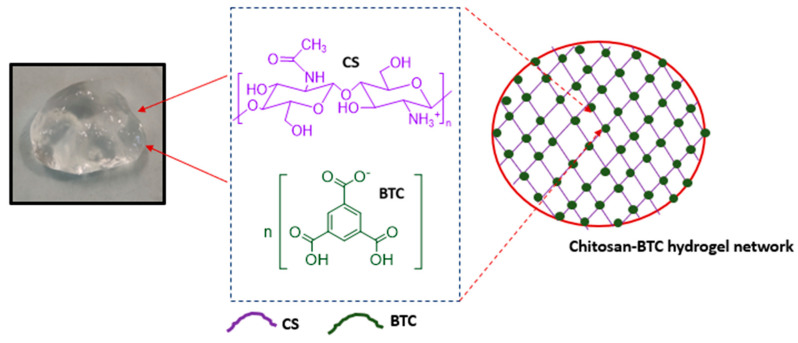
Chitosan–BTC hydrogel (M1).

**Figure 4:**
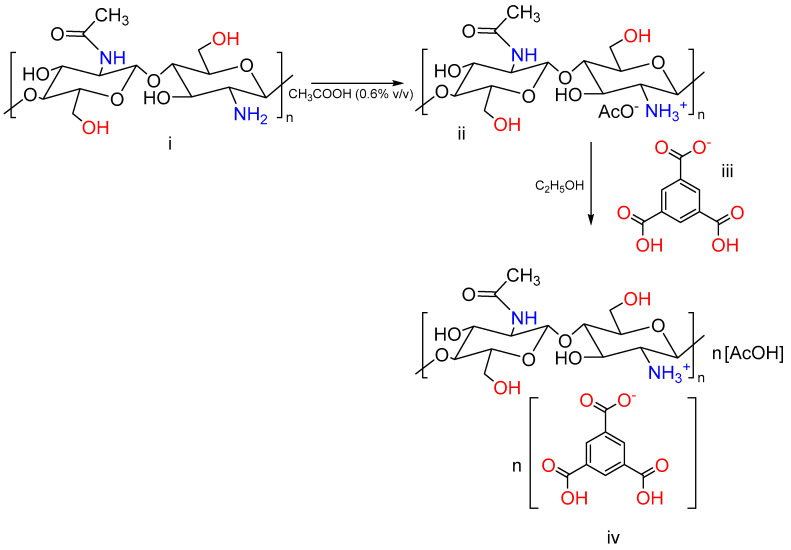
Schematic representation of the cross-linking effect of BTC in the formation of chitosan hydrogels: (**i**) chitosan; (**ii**) protonated chitosan; (**iii**) BTC; and (**iv**) chitosan glucosamine carboxylate salt.

Chitosan hydrogels were prepared by mixing different ratios of chitosan and BTC. Following a visual assessment, no differences in the appearance of the samples (M1–M3) were observed; a picture of M1 is reported in Figure 3 for illustrative purposes. The reactive amino groups on the chitosan can be oriented in the acetic acid solution and form hydrophilic active sites [66]. Then, BTC was dissolved in ethanol and used as cross-linking reagent to form the hydrogel. The interactions between carboxylic acids and chitosan were previously reported in the literature [67]. ^1^H NMR analysis of chitosan–BTC was carried out using DCl/D_2_O [68]. The neutralization reaction between chitosan and BTC can be seen in Figure 4. 

The DDA was calculated using integrals of the proton peaks of the deacetylated monomer (H-D, 3.10 ppm) and protons of the acetyl group (H-A_c_, 1.98 ppm) as proposed by Shigemasa et al. [69]. The DDA of chitosan was calculated as 68.84%. The solvent peak resonated at 4.80 ppm. The small peaks at 1.9 ppm, in both spectra of structures i and iv of Figure 4, originated from the acetyl protons of chitosan. The area between 3 and 4 ppm represents the proton peaks of the deacetylated monomers [70,71]. Furthermore, the singlet at 8.52 ppm confirms the aromatic signals from BTC that do not belong to the structure of chitosan.

### 3.2. Nuclear Magnetic Resonance (NMR) Spectroscopy 

In relation to the chemical structures presented in Figure 4, NMR characterization was conducted, and results are as follows:

Chitosan (i): ^1^H NMR (400 MHz, D_2_O) δ 4.71 ppm (s, 24H), 3.75 ppm (d, *J* = 66.9 Hz, 5H), 3.10 ppm (s, 1H), 1.98 ppm (s, 1H).

BTC (iii): ^1^H NMR (400 MHz, DMSO) δ 13.52 ppm (3H, s), 8.63 ppm (3H, s).

Chitosan glucosamine carboxylate salt (iv): ^1^H NMR (400 MHz, D_2_O) δ 8.52 ppm (s, 5H), 3.65 ppm (d, *J* = 80.0 Hz, 28H), 3.02 ppm (s, 6H), 1.90 ppm (s, 4H).

### 3.3. Computational Study

**Figure 5:**
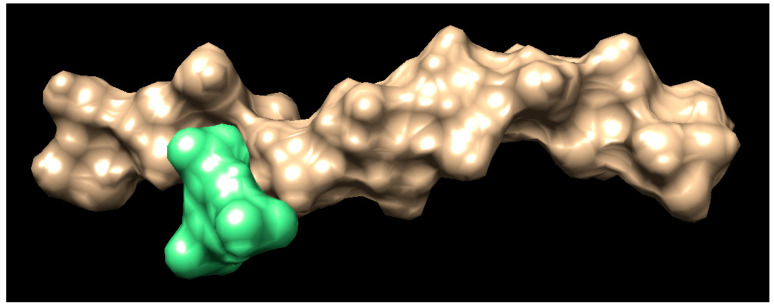
Docking pose (brown, chitosan; green, BTC docked).

To further elucidate the interaction between the polymer (chitosan) and the cross-linked (BTC), a computational study was conducted. Docking is a computational procedure that helps in predicting the binding of one molecule to the pocket of another molecule [72]. This virtual-aided drug design can verify the library of compounds and elucidate the results using a scoring function. This technique is used in the identification of molecular properties using 3D structures [73]. The SMILES string for the chitosan and BTC were obtained using SwissADME software. Below is the SMILES string for the chitosan COC(=O)NC7C(O)C(OC6OC(CO)C(OC5OC(CO)C(OC4OC(CO)C(OC3OC(CO)C(OC2OC(CO)C(OC1OC(CO)C(O)C(O)C1N)C(O)C2N)C(O)C3N)C(O)C4N)C(O)C5N)C(O)C6N)C(CO)OC7OC8C(O)C(N)C(OC8CO)OC9C(O)C(N)C(O)OC9CO. The SMILES string for the BTC is OHC(=O) C1=CC(=CC(=C1) C(OH)=O) C(OH)=O. A table of docking scores appears in a small window, with the tightest binding docking pose for the ligand at the top. The docking poses between chitosan (brown) and BTC (green) can be seen in Figure 5.

REMARK VINA RESULT: −3.3 0.267 4.922

**Figure 6:**
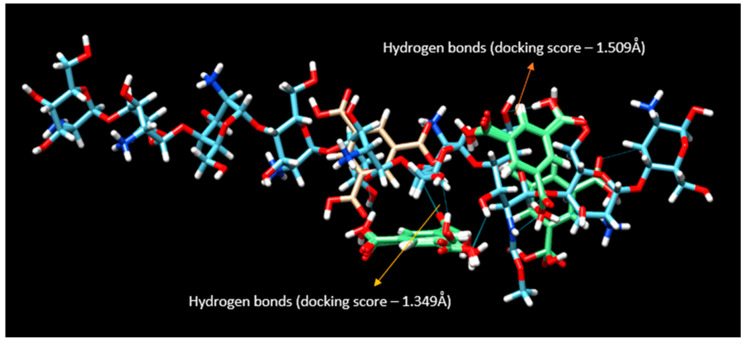
Chimera model. The binding energy of two different torsions (one having a score of −3.4 and another −3.3) are presented in this picture. The two green portions show possible binding of the polymer with BTC.

In the above-represented docking studies (Figure 6), the amine groups of the chitosan interacted with BTC by forming hydrogen bonds. The top ten binding poses from the docking video are remarkably similar in binding energy, i.e., several molecules of carboxylic acid groups were binding to the chitosan at the same time. Hence, all the interactions seem possible from the docking. Many of these interactions can separate the solvent (water) post-gelling. The docking suggests the intermolecular hydrogen bonding between the chitosan and carboxylic acid groups. In addition, there is a possibility of hydrogen bond formation between the carboxylic groups of BTC. The predicted binding energy for each conformation will be given as a docking score in kcal/mole. The two variables in the table are root mean square deviation (RMSD) l.b. (lower bound) and u.b. (upper bound). The docking scores predict the binding affinities of the two molecules once they are docked. The lower the RMSD score, the higher the precision of docking. A negative docking score corresponds to strong binding, and a less negative corresponds to weak binding of the polymer with the docking molecule. From the VINA result, 3.3 kcal/mole is the best binding score with lower bound limit of 0.267 Å and upper bound limit of 4.922 Å. The results also indicate that hydrogen bonding contributes significantly to the interactions between the polymer and the cross-linker.

### 3.4. Fourier nTransform Infrared Spectroscopy (FTIR)

**Figure 7:**
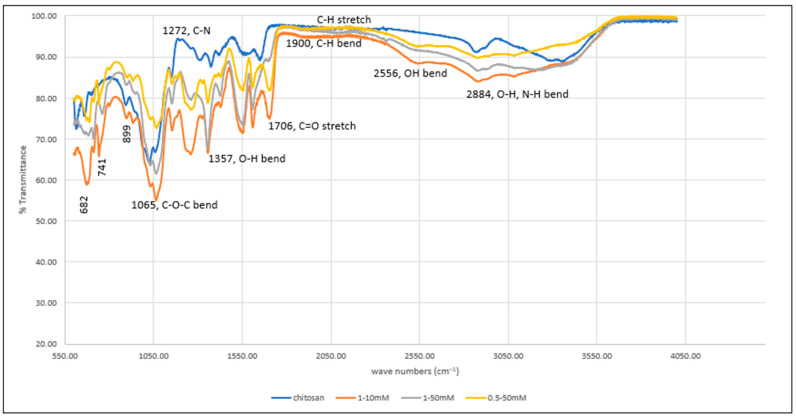
FTIR spectrum of uncross-linked and cross-linked chitosan hydrogel (in different concentrations).

The FTIR spectrum of not cross-linked and of cross-linked hydrogels can be seen in Figure 7. The spectrum shows a band of 2884 cm^−1^ due to O-H and N-H bending (both overlapping in the same region). The band at 2556 cm^−1^ corresponds to the bending of the OH group of the carboxylic acid group (BTC), which cannot be seen in the uncross-linked chitosan hydrogel. In addition, weak C-H bending of the aromatic ring of BTC can be seen in the cross-linked gels. The bands at 1357 cm^−1^ and 1272 cm^−1^ are due to O-H and C-N bending vibrations. The band at 1429 cm^−1^ can be seen in both cross-linked and uncross-linked gels. The strong absorption band at 1549 cm^−1^ corresponds to amide bonds of chitosan. Another band at 1626 cm^−1^ can be due to amine N-H symmetrical vibration [74]. The hydrogels of concentrations 1–10 mM show greater absorption when compared to other concentrations. The carbonyl stretches (C=O) in less concentrated hydrogels showed more prominent peaks when compared to those having higher concentrations at 1706 cm^−1^ and further confirms the presence of BTC in the structure. The strong bending at 682, 741, and 899 cm^−1^ is attributed to aromatic C-H bending, which became less intense at increased concentrations. The C-O-C glycosidic linkage from the chitosan can be seen at 1065 cm^−1^. The interaction between chitosan and BTC can also be proven from the presence of C-O-C bend in the cross-linked signals. These interpretations prove effective cross-linking of chitosan with BTC that takes place at the amino group of chitosan.

### 3.5. Viscosity

The effect of the cross-linker on the polymer concentrations are listed in Table 1.

The values for the torque were recorded between 10 and 100%, and those for the viscosity of the hydrogels were recorded at different shear rates (rpm). An increase in shear rate makes the fluid layers slide over one another at high speed and influences the viscosity of the material. The viscosity of all the formulations decreased with an increase in shear rate which confirms the pseudoplastic behavior of the fluids. Pseudoplasticity is a characteristic feature of shear-thinning fluids and is time-independent. Shear thinning behavior of hydrogel systems are great for biomedical applications. The non-Newtonian behavior is related to the structural reformation of molecules due to flow. High shear rates can cause the breakdown of fluid structures leading to reduced viscosity. This property influences the performance of the hydrogel during drug delivery via injection [75]. In addition, the viscosity of sample M1 is higher than other concentrations. The high viscosity of formulations often affects the injectability and syringeability performance while injecting the hydrogel [76]. Presumably, the viscosity data shows greater and more efficient cross-linking at lower BTC concentrations as opposed to high concentrations.

### 3.6. Texture Analysis

Table 2 reports the results of texture analysis of the hydrogels at three different concentrations (M1, M2, and M3). The instrument can measure the forward and backward extrusions and present the recorded forces (A and B) in response to the contraction and retraction of the probe. Initially, the instrument was calibrated for force and height measurement. The probe was programmed as per the optimized test conditions, and measurements were taken. 

**Figure 8:**
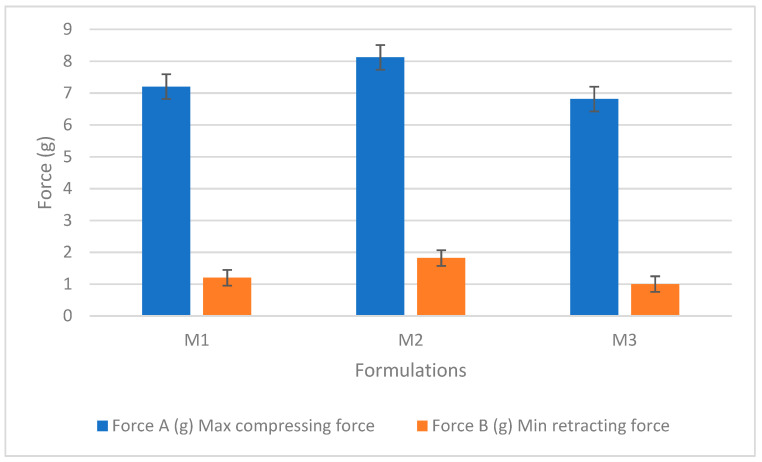
The compressing and retracting forces of the three hydrogel formulations (M1, M2, and M3). Results represent mean values ± SD. n = 3.

During the test, the probe traveled at a speed of 2 mm/s downwards into the gel and was then withdrawn. Gel parameters, including hardness, cohesiveness, and adhesiveness, were evaluated using a standard force-time plot (as seen in Figure S7). The readings were taken in triplicates. The variables from the table show retracting, compressing, cohesiveness, and adhesiveness, which cumulatively determine the texture of the material. Area A1 shows the cohesiveness of the gel, while area B1 shows the adhesiveness of the hydrogel to the probe [61]. Cohesiveness is a feature where particles stick to each other and influence the flow properties. An increase in cohesiveness causes a decrease in the flow due to agglomeration of the gel molecules [77]. In Table 2, M2 shows high cohesiveness, hence retarding the flow of the hydrogels. The adhesiveness of the gels was represented by the retracting force B1. This is the work required to overcome the forces between the surface of the gel and the probe [78]. The cohesiveness and adhesiveness of different concentrations of gels were plotted against the area as shown in Figure 8. Though the difference in adhesiveness is not significant, a slight increase is observed for M2, which might be attributed to the higher ability of the gel to chemically interact with the probe. The hardness of the gels was measured from the maximum compressing force of the gel formulations. It gives the force required to deform the gels. The hydrogel’s hardness values can be verified based on the area of the application. The hydrogel concentrations with low compressing force showed less cohesiveness.

### 3.7. Oscillation Amplitude Strain Sweep Experiments

**Figure 9:**
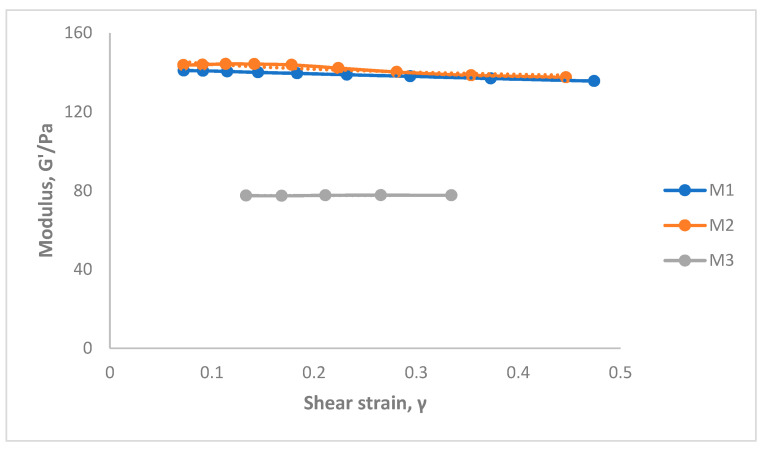
Amplitude strain sweep for hydrogels prepared with different concentrations of chitosan–BTC. (See Supplementary Tables S2–S4).

The oscillation amplitude strain tests provide information regarding the effect of BTC and polymer concentration on the sample structures. The influence of stress amplitude on the hydrogels can be seen in Figure 9. The tests were conducted under constant stress and strain and help to determine the hydrogels’ viscoelastic properties. Strain sweep tests confirmed the gel-like behavior since the data of all the samples showed the elastic (storage) modulus G′ higher than the loss (viscous) modulus G″ (as shown in the figures). In addition, the flow properties of the gels can be determined from the complex modulus (G*) and the phase angle (δ). The complex modulus determines the stiffness of the material.

The LVE region of the hydrogels can be seen in Figures S8–S10. This region implies a stress range over which G′ is independent of applied shear stress. For evaluating the mechanical strength of the hydrogels, the elastic modulus from the LVE region was compared with the strain values. Hydrogels with less amount of chitosan (M3) have the elastic modulus value of 77.15 Pa when compared to high G′ 135.7 Pa (M2) and medium G′ of 134.7 Pa (M1). The high level of polymer in the cross-linked network has given a stronger hydrogel when compared to low levels of chitosan and BTC. From the above high and medium G′ values, we can see little difference due to the comparable concentrations of the polymer in both samples (M1 and M2). In case of M2, the G′ values decreased abruptly at a shear strain of 0.5%. This evidence suggests cross-linking of these hydrogels resulted in the following observations: (1) an increase of elastic modulus of G′ that refers to the material deformation, i.e., of the intermolecular networks in the gel structure; and (2) an increase in shear stress is observed in M2 in the linear viscoelastic region.

The rheological data from the oscillation tests provided the calculated shear stress and strain as seen in Table 3. In theory, the shear modulus is defined as the ratio of shear stress to the shear strain. When the shear modulus of the material is higher than the other, then the material is known to have high rigidity. The shear modulus of the hydrogels with M2 was found to be higher than other samples. Therefore, the material is found to have high rigidity.

### 3.8. Frequency Strain Sweep Experiments

**Figure 10:**
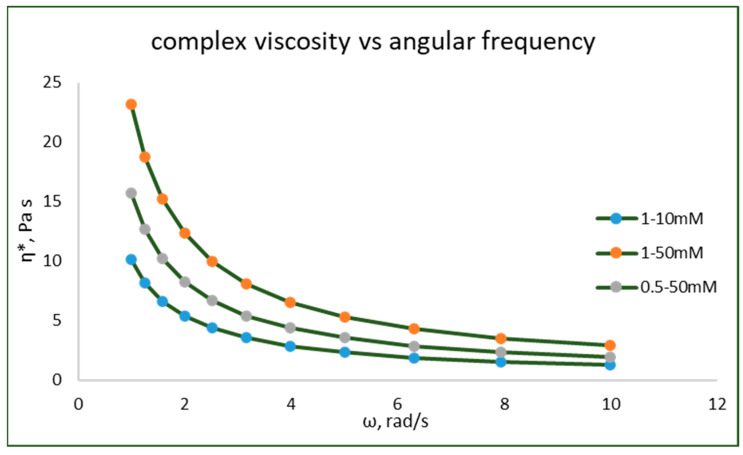
Frequency sweep (ω) and complex viscosity (η) of hydrogels at different concentrations. (See Supplementary Tables S5–S7).

Frequency strain sweep experiments for the hydrogels were performed within the LVE region to determine the frequency dependence. The distribution of frequencies in Tables S5–S7 clearly shows the dispersion and association of particles in the hydrogel structure. The stability of the cross-linked networks can be studied using these tests. Figure 10 represents the graph plotted between the elastic modulus and the frequency. The angular frequency of the test material was set as ranging between 0.1 and 10 rad/s. Both G′ and G″ were frequency-dependent, which can be attributed to the viscoelastic properties of the hydrogel network. At a high frequency of 10 Hz, the shear viscosity of M1 was 2.9 Pa s, which increased with increasing concentrations of both polymer and cross-linker. 

Interestingly, with an increase in complex viscosity, the angular frequency of the hydrogels decreased. This confirms that the hydrogels show a shear-thinning behavior, thus proving that the hydrogels prepared were pseudoplastic fluids. In addition, the elastic modulus G′ of the hydrogels was higher than the loss modulus G″ and thus confirms the behavior.

### 3.9. Scanning Electron Microscopy

**Figure 11:**
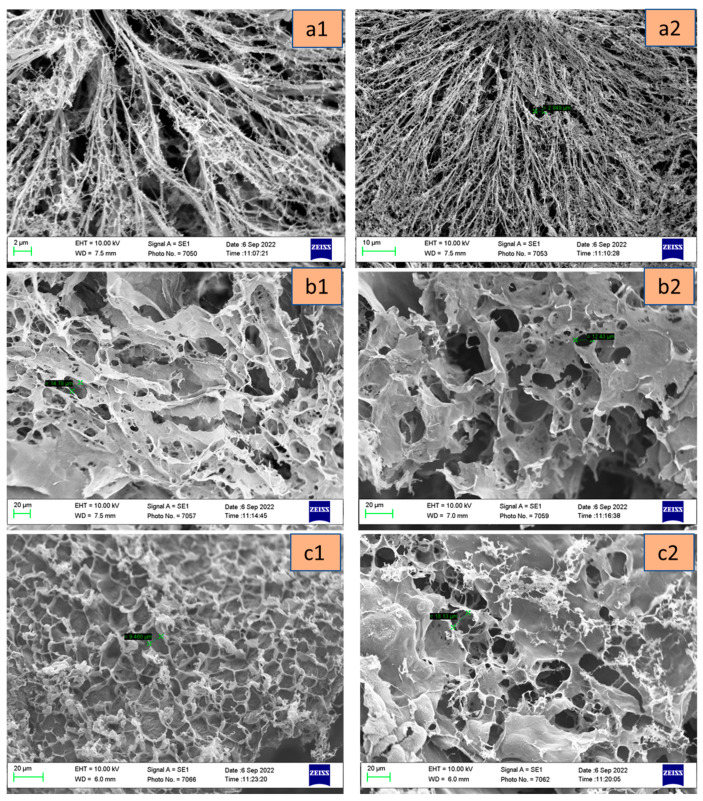
SEM structures of freeze-dried chitosan hydrogels M1 (**a1**,**a2**), M2 (**b1**,**b2**), and M3 (**c1**,**c2**) at different magnifications.

The scanning electron microscopy (SEM) images of chitosan–BTC gels demonstrated the presence of interconnected pores between the gel networks as seen in Figure 11. The lyophilized gels have highly connected pores, which can allow the passage of nutrients and drugs to the site of action. The gels with concentrations M1, M2, and M3 have pore size ranging from 2.8–3.3 µm, 14–16 µm, and 5–18 µm, respectively. Here, it can be noted that hydrogels with low concentrations of BTC present smaller pore sizes when compared to higher concentrations. The higher the porosity, the higher the rate of drug release [79]. M1 with low BTC depicts long streaks of branched out polymer network, while M2 and M3 with relatively high BTC appear to have formed more tortuous pores. So, from the previous observations, the hydrogels with higher BTC concentrations were found suitable for drug delivery applications. 

### 3.10. Drug Release Studies

**Figure 12:**
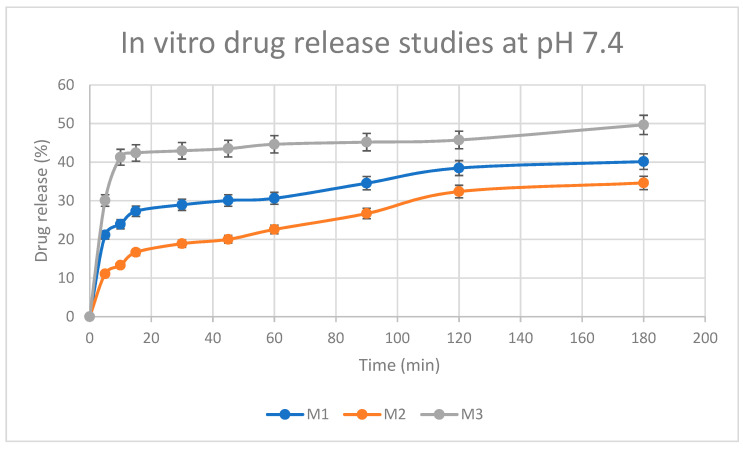
Release profile of 5-FU incorporated in chitosan–BTC hydrogels (M1, M2, and M3) investigated in phosphate-buffered saline (PBS) medium, pH 7.4. Results represent mean values ± SD; n = 3 (For M1 and M2: *p* > 0.05; M1 and M3: *p* < 0.05).

Figure 12 shows the in vitro release behavior of the three samples (namely: M1, M2, and M3) of BTC-cross-linked chitosan hydrogels loaded with 5-FU. The rate of drug release from the hydrogels was noted to be more pronounced in the first 20 min, followed by a more gradual release, amounting to an overall release between 35% and 50% within the study period of 180 min [39,54]. The amino groups of chitosan are not protonated at pH 7.4, resulting in the formation of physical networks in the hydrogels. These networks are responsible for the controlled release of the drug in PBS medium as reported in the literature [80]. In addition, the degree of cross-linking of the polymer influenced the release capacity of the hydrogel matrix. In general, high levels of cross-linker improve drug loading effectiveness, while slowing the rate at which entrapped drugs are released. Interestingly, the concentrations of chitosan and BTC influenced the pattern of 5-FU release from the hydrogel matrix. The drug release profiles of chitosan–BTC at 37 °C can be seen in Figure 12. A higher concentration of BTC, which in turn results in a greater extent of cross-linking in M2, caused a retarded drug release in the first 60 min. On the other hand, when chitosan concentration was reduced from 1% to 0.5% (i.e., comparing M2 with M3), a ‘burst-effect’ in drug release was noted within the first 20 min. A lower amount of chitosan in the M3 hydrogel, yielding less cross-linking with BTC, may have enabled more pronounced drug release. According to ANOVA, statistically significant differences in drug release were observed between hydrogels with different chitosan/BTC ratios. In the case of M1 and M2, the *t*-test (*p* = 0.21) indicates no significant difference in time required for drug release. However, there is a significant difference in time for drug release between M2 and M3 (*p* = 0.007). Based on these studies, the prepared hydrogel can be used for drug delivery systems and other biomedical applications.

**Figure 13:**
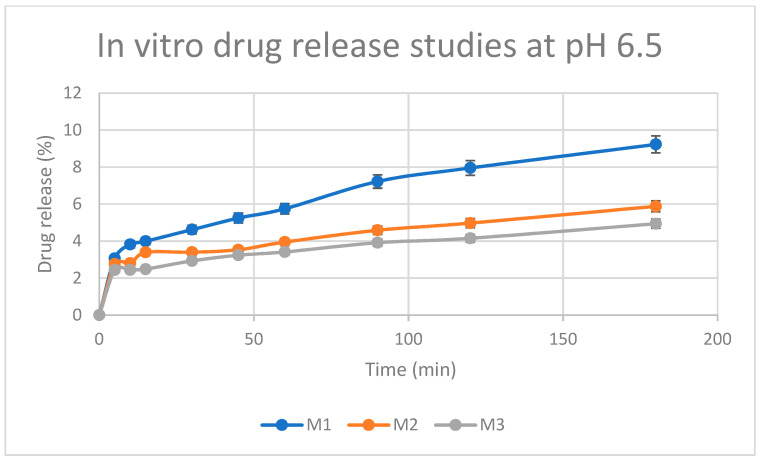
Release profile of 5-FU incorporated in chitosan–BTC hydrogels (M1, M2, and M3) investigated in phosphate-buffered saline (PBS) medium, pH 6.5 Results represent mean values ± SD; n = 3 (For M1 and M2: *p* < 0.05; M1 and M3: *p* < 0.05).

At pH 6.5, rapid release of the drug occurs from 0 to 5 min, and then the drug is released gradually from 5 to 180 min (as seen in Figure 13). In addition, there is not a significant difference in time for drug release between the gel concentrations (M1, M2, and M3) at this pH.

When comparing the 2 release profiles at different pH values, it is evident that at pH = 7.4 there is a higher extent of drug (cumulative) release than at pH 6.5. This might suggest that 5-FU is not released via erosion of the hydrogel, (i.e., the polymer is solubilized, and the hydrogel is disassembled); 5-FU is rather released via diffusion which is favoured in non-acidic environments.

## 4. Conclusions

The current research aimed to develop BTC-cross-linked chitosan hydrogels containing 5-FU for cancer therapy. Using different ratios of BTC/chitosan, flow curves of the hydrogels revealed shear thinning behavior of the resulting hydrogels. With an increase in the shear rate, there is a decline in the apparent viscosity, which is a characteristic feature of shear-thinning fluids. Viscoelastic investigations of the hydrogels revealed the elastic (G′) modulus values were higher than the viscous (G″) values, hence confirming the elastic behavior of all prepared hydrogels. Further oscillatory tests (strain and frequency sweep) confirmed the stable hydrogels’ behavior since all exhibited a plateau in the range of 0.1–10 Hz. Under physiological conditions, the gel behaviour can be tuned by changing the cross-linker concentrations. The low G′ (77.15 Pa) and G″ (8.711 Pa) values of M2 indicate poor mechanical strength when compared to other samples. Spectroscopic and structural investigations of the formulated hydrogels (NMR, FTIR, and SEM) confirmed the presence of glycosidic bonds signals in all the spectra. The cross-sectional freeze-dried images exhibited porous, compact, and homogenous distribution of hydrogel networks. This provides a scope for encapsulating drugs in the hydrogel matrix. The hydrogels exhibited slow drug release in PBS (<50%) that provides a scope for sustained drug release. Additionally, the release at slightly acidic pH showed a slower and more prolonged release of the drug, which might be explained in light of the porous structure of the hydrogel matrix, coupled with the ionization of chitosan in acidic pH environments. The drug release also appeared to have a direct correlation with the extent of polymer cross-linking, thus facilitating a sustained drug delivery. This is a desired characteristic of hydrogels which tends to enhance the safety and efficacy of drug therapy. 

Overall, the data from this study on the properties of hydrogels and their behavior in vitro demonstrates a great potential for enhanced biological performance and thus warrant further investigations.

## Figures and Tables

**Table 1:** Viscosities of hydrogels with different concentrations (M1, M2, and M3).

Samples	Viscosity	10 rpm	Viscosity	20 rpm	Viscosity	50 rpm	Viscosity	60 rpm	Viscosity	100 rpm
	Torque (%)	cP	Torque (%)	cP	Torque (%)	cP	Torque (%)	cP	Torque (%)	cP
M1	11.4	135.6	13.5	81	14.7	43.5	17.5	21	21.3	12.7
M2	11.1	66.6	12.8	38	14	16.6	15.6	15.6	17	10.2
M3	10.2	57	10.5	32.4	13.3	16	14	14	17.4	10.4

**Table 2:** Texture properties of the hydrogel formulations under the optimized test conditions.

	Force A1 (g)	Area A1 (g*sec)	Force B1 (g)	Area B1 (g*sec)
Sample	(Max. Compressing Force, Hardness)	Cohesiveness	Min. Retracting Force	Adhesiveness
M1	7.201	6.717	1.200	1.466
M2	8.120	16.348	1.820	3.321
M3	6.810	6.073	1.000	2.142

**Table 3:** The calculated shear stress and strain results from within the LVE range.

Sample	Shear Stress (Pa)	Shear Strain	Shear Modulus
M1	6.4297 × 10^−2^	4.7421 × 10^−2^	1.35587609
M2	2.033 × 10^−1^	1.4887 × 10^−1^	1.36562101
M3	2.5966 × 10^−2^	3.344 × 10^−2^	7.76495215 × 10^−1^

## Data Availability

Data are available upon request.

## Supplementary Materials

The following supporting information can be downloaded at: https://www.mdpi.com/article/10.3390/pharmaceutics15041084/s1, Figure S1: ^1^H NMR spectra of chitosan (i). Figure S2. 1H NMR spectra of BTC (iii). Figure S3. 1H NMR spectra of chitosan glucosamine carboxylate salt (iv). Figure S4. IR spectra of M1 hydrogel. Figure S5. IR of M2 hydrogel. Figure S6. IR spectra of M3 hydrogel. Figure S7. Force vs. time plot of chitosan–BTC hydrogels in three different concentrations: M1, M2, and M3. Figure S8. Oscillation dynamics of elastic and viscous moduli of chitosan cross-linked with BTC (M1 hydrogel). Figure S9. Oscillation dynamics of elastic and viscous moduli of chitosan cross-linked with BTC (M2 hydrogel). Figure S10. Oscillation dynamics of elastic and viscous moduli of chitosan cross-linked with BTC (M3 hydrogel). Table S1. Hydrogels compositions. Table S2. Amplitude strain sweep for hydrogels with chitosan–BTC concentration 1–10 Mm (sup-plementary material). Table S3. Amplitude strain sweep for hydrogels with chitosan–BTC concentration 1–50 mM (sup-plementary material). Table S4. Amplitude strain sweep for hydrogels with chitosan–BTC concentration 0.5–50 Mm (supplementary material). Table S5. Frequency sweep for hydrogels with chitosan–BTC concentration 1–10 mM (supple-mentary material). Table S6. Frequency strain sweep for hydrogels with chitosan–BTC concentration 1–50 mM (supplementary material). Table S7. Frequency strain sweep for hydrogels with chitosan–BTC concentration 0.5–50 mM (supplementary material). Table S8. Frequency strain sweep for hydrogels with chitosan–BTC concentration 0.5–50 mM (supplementary material).

## References

[1] Ahmed E.M. (2015). Hydrogel: Preparation, characterization, and applications: A review. J. Adv. Res..

[2] Sharpe A.L., Daily A.M., Horava S.D., A Peppas N. (2014). Therapeutic applications of hydrogels in oral drug delivery. Expert Opin. Drug Deliv..

[3] Narayanaswamy R., Torchilin V.P. (2019). Hydrogels and their applications in targeted drug delivery. Molecules.

[4] Ishihara M., Obara K., Nakamura S., Fujita M., Masuoka K., Kanatani Y., Takase B., Hattori H., Morimoto Y., Ishihara M. (2006). Chitosan hydrogel as a drug delivery carrier to control angiogenesis. J. Artif. Organs.

[5] Moeinzadeh S., Park Y., Lin S., Yang Y.P. (2020). In-situ stable injectable collagen-based hydrogels for cell and growth factor delivery. Materialia.

[6] Yu Z., Li H., Xia P., Kong W., Chang Y., Fu C., Wang K., Yang X., Qi Z. (2020). Application of fibrin-based hydrogels for nerve protection and regeneration after spinal cord injury. J. Biol. Eng..

[7] Zainal S.H., Mohd N.H., Suhaili N., Anuar F.H., Lazim A.M., Othaman R. (2021). Preparation of cellulose-based hydrogel: A review. J. Mater. Res. Technol..

[8] Xu X., Jha A.K., Harrington D.A., Farach-Carson M.C., Jia X. (2012). Hyaluronic acid-based hydrogels: From a natural polysaccharide to complex networks. Soft Matter.

[9] Oh Y., Cha J., Kang S.-G., Kim P. (2016). A polyethylene glycol-based hydrogel as macroporous scaffold for tumorsphere formation of glioblastoma multiforme. J. Ind. Eng. Chem..

[10] Jensen B.E.B., Dávila I., Zelikin A.N. (2016). Poly(vinyl alcohol) physical hydrogels: Matrix-mediated drug delivery using spontaneously eroding substrate. J. Phys. Chem. B.

[11] Sharifzadeh G., Hezaveh H., Muhamad I.I., Hashim S., Khairuddin N. (2019). Montmorillonite-based polyacrylamide hydrogel rings for controlled vaginal drug delivery. Mater. Sci. Eng. C.

[12] Qiu Y., Park K. (2001). Environment-Sensitive Hydrogels for Drug Delivery. www.elsevier.com/locate/drugdeliv.

[13] Berkovitch Y., Seliktar D. (2017). Semi-synthetic hydrogel composition and stiffness regulate neuronal morphogenesis. Int. J. Pharm..

[14] Rossi B., Venuti V., D’Amico F., Gessini A., Mele A., Punta C., Melone L., Crupi V., Majolino D., Masciovecchio C. (2016). Guest-matrix interactions affect the solvation of cyclodextrin-based polymeric hydrogels: A UV Raman scattering study. Soft Matter.

[15] Concheiro A., Alvarez-Lorenzo C. (2013). Chemically cross-linked and grafted cyclodextrin hydrogels: From nanostructures to drug-eluting medical devices. Adv. Drug Deliv. Rev..

[16] Naveed M., Phil L., Sohail M., Hasnat M., Baig M.M.F.A., Ihsan A.U., Shumzaid M., Kakar M.U., Khan T.M., Akabar M.D. (2019). Chitosan oligosaccharide (COS): An overview. Int. J. Biol. Macromol..

[17] Kim J.H., Lee J.-H., Kim K.-S., Na K., Song S.-C., Lee J., Kuh H.-J. (2013). Intratumoral delivery of paclitaxel using a thermosensitive hydrogel in human tumor xenografts. Arch. Pharmacal Res..

[18] Pesoa J.I., Rico M.J., Rozados V.R., Scharovsky O.G., Luna J.A., Mengatto L.N. (2018). Paclitaxel delivery system based on poly(lactide-co-glycolide) microparticles and chitosan thermosensitive gel for mammary adenicarcinoma treatment. J. Pharm. Pharmacol..

[19] Zhang N., Xu X., Zhang X., Qu D., Xue L., Mo R., Zhang C. (2016). Nanocomposite hydrogel incorporating gold nanorods and paclitaxel-loaded chitosan micelles for combination photothermal chemotherpay. Int. J. Pharm..

[20] Jiang Y., Meng X., Wu Z., Qi X. (2016). Modified chitosan thermosensitive hydrogel enables sustained and efficient anti-tumor therapy via intratumoral injection. Carbohydr. Polym..

[21] Ishihara M., Fujita M., Obara K., Hattori H., Nakamura S., Nambu M., Kiyosawa T., Kanatani Y., Takase B., Kikuchi M. (2006). Controlled releases of FGF-2 and Paclitaxel from Chitosan Hydrogels and their Subsequent Effects on Wound Repair, Angiogenesis and Tumor Growth. Curr. Drug Deliv..

[22] Liu J., Zhang L., Yang Z., Zhao X. (2011). Controlled release of paclitaxel from a self-assembling peptide hydrogel formed insitu and antitumor study in vitro. Int. J. Nanomed..

[23] Croisier F., Jérôme C. (2013). Chitosan-based biomaterials for tissue engineering. Eur. Polym. J..

[24] Bandara S., Du H., Carson L., Bradford D., Kommalapati R. (2020). Agricultural and biomedical applications of chitosan-based nanomaterials. Nanomaterials.

[25] Elieh-Ali-Komi D., Hamblin M.R., Daniel E.-A.-K. (2016). Chitin and Chitosan: Production and Application of Versatile Biomedical Nanomaterials. Int. J. Adv. Res..

[26] Hamedi H., Moradi S., Hudson S.M., Tonelli A.E. (2018). Chitosan based hydrogels and their applications for drug delivery in wound dressings: A review. Carbohydr. Polym..

[27] Safer A.-M., Leporatti S. (2021). Chitosan Nanoparticles for Antiviral Drug Delivery: A Novel Route for COVID-19 Treatment. Int. J. Nanomed..

[28] Cao Y., Tan Y.F., Wong Y.S., Liew M.W.J., Venkatraman S. (2019). Recent advances in chitosan-based carriers for gene delivery. Mar. Drugs.

[29] Sandri S.R.G., Bonferoni M., Ferrari F., Mori M., Caramella C. (2012). The role of chitosan as a mucoadhesive agent in mucosal drug delivery. J. Drug Deliv. Sci. Technol..

[30] Aranaz I., Alcántara A.R., Civera M.C., Arias C., Elorza B., Caballero A.H., Acosta N. (2021). Chitosan: An overview of its properties and applications. Polymers.

[31] Snoreen S., Pervaiz F., Ashames A., Buabeid M., Fahelelbom K., Shoukat H., Maqbool I., Murtaza G. (2021). Optimization of novel naproxen-loaded chitosan/carrageenan nanocarrier-based gel for topical delivery: Ex vivo, histopathological, and in vivo evaluation. Pharmaceuticals.

[32] Beppu M., Vieira R., Aimoli C., Santana C. (2007). Crosslinking of chitosan membranes using glutaraldehyde: Effect on ion permeability and water absorption. J. Membr. Sci..

[33] Monteiro O.A., Airoldi C. (1999). Some studies of crosslinking chitosan-glutaraldehyde interaction in a homogeneous system. Int. J. Biol. Macromol..

[34] Kildeeva N.R., Perminov P.A., Vladimirov L.V., Novikov V.V., Mikhailov S.N. (2009). About mechanism of chitosan cross-linking with glutaraldehyde. Russ. J. Bioorganic Chem..

[35] Vo N.T.N., Huang L., Lemos H., Mellor A.L., Novakovic K. (2021). Genipin-crosslinked chitosan hydrogels: Preliminary evaluation of the in vitro biocompatibility and biodegradation. J. Appl. Polym. Sci..

[36] Muzzarelli R.A. (2009). Genipin-crosslinked chitosan hydrogels as biomedical and pharmaceutical aids. Carbohydr. Polym..

[37] Jayakumar R., Reis R.L., Mano J.F. (2006). Phosphorous containing chitosan beads for controlled oral drug delivery. J. Bioact. Compat. Polym..

[38] Kaur K., Paiva S.S., Caffrey D., Cavanagh B.L., Murphy C.M. (2021). Injectable chitosan/collagen hydrogels nano-engineered with functionalized single wall carbon nanotubes for minimally invasive applications in bone. Mater. Sci. Eng. C.

[39] Yang Y., Chen G., Murray P., Zhang H. (2020). Porous chitosan by crosslinking with tricarboxylic acid and tuneable release. SN Appl. Sci..

[40] Buschmann M.D., Merzouki A., Lavertu M., Thibault M., Jean M., Darras V. (2013). Chitosans for delivery of nucleic acids. Adv. Drug Deliv. Rev.

[41] Larrañeta E., Stewart S., Ervine M., Al-Kasasbeh R., Donnelly R.F. (2018). Hydrogels for Hydrophobic Drug Delivery. Classification, Synthesis and Applications. J. Funct. Biomater..

[42] Vashist A., Ahmad S. (2013). Hydrogels: Smart Materials for Drug Delivery. Orient. J. Chem..

[43] Bhatt R., Sreedhar B., Padmaja P. (2017). Chitosan supramolecularly cross linked with trimesic acid—Facile synthesis, characterization and evaluation of adsorption potential for chromium(VI). Int. J. Biol. Macromol..

[44] Tang L.M., Wang Y.J. (2009). Highly stable supramolecular hydrogels formed from 1,3,5-benzenetricarboxylic acid and hydroxyl pyridines. Chin. Chem. Lett..

[45] Goudoulas T.B., Germann N. (2017). Phase transition kinetics and rheology of gelatin-alginate mixtures. Food Hydrocoll..

[46] Pai V., Srinivasarao M., Khan S.A. (2002). Evolution of microstructure and rheology in mixed polysaccharide systems. Macromolecules.

[47] Richa, Choudhury A.R. (2018). Synthesis and rheological characterization of a novel thermostable quick setting composite hydrogel of gellan and pullulan. Int. J. Biol. Macromol..

[48] Zuidema J.M., Rivet C.J., Gilbert R.J., Morrison F.A. (2013). A protocol for rheological characterization of hydrogels for tissue engineering strategies. J. Biomed. Mater. Res. Part B Appl. Biomater..

[49] Malvern Panalytical (2023). Rheological Analysis of Dispersions by Frequency Sweep Testing.

[50] Ferry J.D. (1980). Viscoelastic Properties of Polymers.

[51] Matricardi P., Dentini M., Crescenzi V. (1987). Porphyrin Amphiphiles as Templates for the Nucleation of Calcium Carbonate. https://pubs.acs.org/sharingguidelines.

[52] Montembault A., Viton C., Domard A. (2005). Rheometric Study of the Gelation of Chitosan in Aqueous Solution without Cross-Linking Agent. Biomacromolecules.

[53] Laukkanen O.-V., Winter H.H. (2017). Strain accumulation in bituminous binders under repeated creep-recovery loading predicted from small-amplitude oscillatory shear (SAOS) experiments. Mech. Time-Depend. Mater..

[54] Gull N., Khan S.M., Butt M.T.Z., Khalid S., Shafiq M., Islam A., Asim S., Hafeez S., Khan R.U. (2019). In vitro study of chitosan-based multi-responsive hydrogels as drug release vehicles: A preclinical study. RSC Adv..

[55] Zhang D.-Y., Shen X.-Z., Wang J.-Y., Dong L., Zheng Y.-L., Wu L.-L. (2008). Preparation of chitosan-polyaspartic acid-5-fluorouracil nanoparticles and its anti-carcinoma effect on tumor growth in nude mice. World J. Gastroenterol..

[56] Hou Q., De Bank P.A., Shakesheff K.M. (2004). Injectable scaffolds for tissue regeneration. J. Mater. Chem..

[57] Lee S.-Y., Tae G. (2007). Formulation and in vitro characterization of an in situ gelable, photo-polymerizable Pluronic hydrogel suitable for injection. J. Control. Release.

[58] Trott O., Olson A.J. (2010). AutoDock Vina: Improving the speed and accuracy of docking with a new scoring function, efficient optimization, and multithreading. J. Comput. Chem..

[59] Pettersen E.F., Goddard T.D., Huang C.C., Couch G.S., Greenblatt D.M., Meng E.C., Ferrin T.E. (2004). UCSF Chimera? A visualization system for exploratory research and analysis. J. Comput. Chem..

[60] Daina A., Michielin O., Zoete V. (2017). SwissADME: A free web tool to evaluate pharmacokinetics, drug-likeness and medicinal chemistry friendliness of small molecules. Sci. Rep..

[61] Jones D.S., Woolfson A., Brown A.F. (1997). Textural, viscoelastic and mucoadhesive properties of pharmaceutical gels composed of cellulose polymers. Int. J. Pharm..

[62] Jones D.S., Andrews G.P., Gorman S.P. (2005). Characterization of crosslinking effects on the physicochemical and drug diffusional properties of cationic hydrogels designed as bioactive urological biomaterials. J. Pharm. Pharmacol..

[63] Hurler J., Engesland A., Kermany B.P., Škalko-Basnet N. (2011). Improved texture analysis for hydrogel characterization: Gel cohesiveness, adhesiveness, and hardness. J. Appl. Polym. Sci..

[64] Noreen S., Pervaiz F., Ijaz M., Shoukat H. (2022). Synthesis and characterization of pH-sensitive chemically crosslinked block copolymer [Hyaluronic acid/Poloxamer 407-co-poly (Methacrylic acid)] hydrogels for colon targeting. Polym. Technol. Mater..

[65] Obata Y., Nishino T., Kushibiki T., Tomoshige R., Xia Z., Miyazaki M., Abe K., Koji T., Tabata Y., Kohno S. (2012). HSP47 siRNA conjugated with cationized gelatin microspheres suppresses peritoneal fibrosis in mice. Acta Biomater..

[66] Shamov M., Bratskaya S., Avramenko V. (2002). Interaction of Carboxylic Acids with Chitosan: Effect of pK and Hydrocarbon Chain Length. J. Colloid Interface Sci..

[67] Mitani T., Yamashita T., Okumura C., Ishii H. (1995). Adsorption of Benzoic Acid and Its Derivatives to Swollen Chitosan Beads. Biosci. Biotechnol. Biochem..

[68] Shapiro Y.E. (2011). Structure and dynamics of hydrogels and organogels: An NMR spectroscopy approach. Prog. Polym. Sci..

[69] Lavertu M., Xia Z., Serreqi A., Berrada M., Rodrigues A., Wang D., Buschmann M., Gupta A. (2003). A validated 1H NMR method for the determination of the degree of deacetylation of chitosan. J. Pharm. Biomed. Anal..

[70] Vårum K.M., Anthonsen M.W., Grasdalen H., Smidsrød O. (1991). Determination of the degree of N-acetylation and the distribution of N-acetyl groups in partially N-deacetylated chitins (chitosans) by high-field n.m.r. spectroscopy. Carbohydr. Res..

[71] Shigemasa Y., Matsuura H., Sashiwa H., Saimoto H. (1996). Biological Macromolecules Evaluation of different absorbance ratios from infrared spectroscopy for analyzing the degree of deacetylation in chitin. Int. J. Biol. Macromol..

[72] Azam S.S., Abbasi S.W. (2013). Molecular docking studies for the identification of novel melatoninergic inhibitors for acetylserotonin-O-methyltransferase using different docking routines. Theor. Biol. Med. Model..

[73] Mani S., Supriya T., Shankar M., Lalitha S.K., Dastgiri J., Babu M.N. (2016). A Over View on Molecular Docking American Journal of Biological and Pharmaceutical Research a over View on Molecular Docking. Am. J. Biol. Pharm. Res..

[74] Altinisik A., Yurdakoç K. (2013). Chitosan/poly(vinyl alcohol) hydrogels for amoxicillin release. Polym. Bull..

[75] Muthu M.S., Rawat M.K., Mishra A., Singh S. (2009). PLGA nanoparticle formulations of risperidone: Preparation and neuropharmacological evaluation. Nanomedicine.

[76] Zhang Q., Fassihi M.A., Fassihi R. (2018). Delivery Considerations of Highly Viscous Polymeric Fluids Mimicking Concentrated Biopharmaceuticals: Assessment of Injectability via Measurement of Total Work Done “WT”. AAPS PharmSciTech.

[77] Tobin A.B., Heunemann P., Wemmer J., Stokes J.R., Nicholson T., Windhab E.J., Fischer P. (2017). Cohesiveness and flowability of particulated solid and semi-solid food systems. Food Funct..

[78] Cevher E., Taha M.A., Orlu M., Araman A. (2008). Evaluation of Mechanical and Mucoadhesive Properties of Clomiphene Citrate Gel Formulations Containing Carbomers and Their Thiolated Derivatives. Drug Deliv..

[79] Varghese J.S., Chellappa N., Fathima N.N. (2014). Gelatin–carrageenan hydrogels: Role of pore size distribution on drug delivery process. Colloids Surf. B Biointerfaces.

[80] Butt A., Jabeen S., Nisar N., Islam A., Gull N., Iqbal S.S., Khan S.M., Yameen B. (2018). Controlled release of cephradine by biopolymers based target specific crosslinked hydrogels. Int. J. Biol. Macromol..

