# Supplementary material for: Chitosan Hydrogels Cross-Linked with Trimesic Acid for the Delivery of 5-Fluorouracil in Cancer Therapy
Source: Pharmaceutics. 2023 Mar 28;15(4):1084. doi: 10.3390/pharmaceutics15041084 (PMC10143928; doi:10.3390/pharmaceutics15041084)
Supplement: Supplementary file 1 [file pharmaceutics-15-01084-s001.zip › pharmaceutics-2192732-supplementary.pdf]

pharmaceutics

MDPI

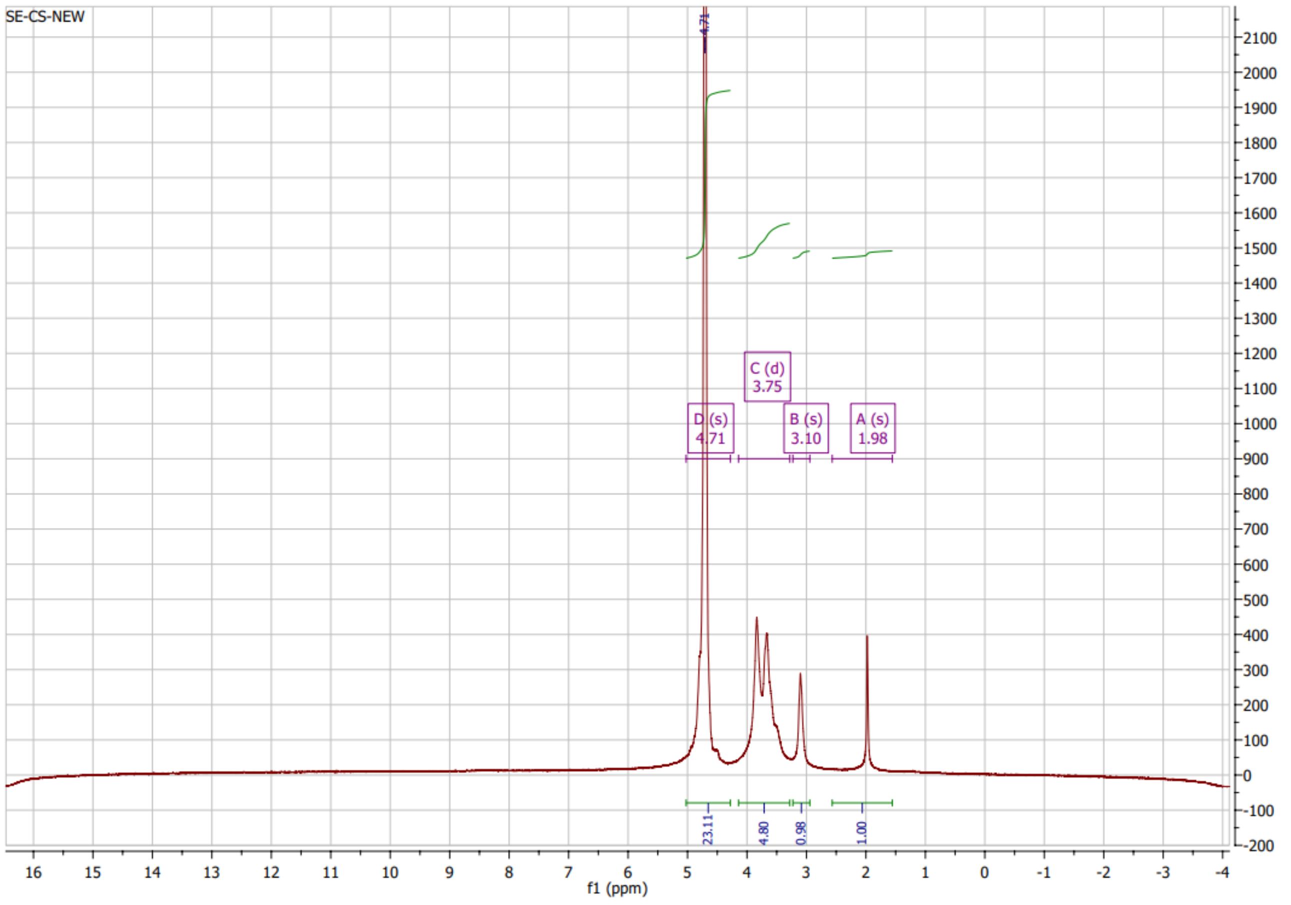

Figure S1. ^{1}H NMR spectra of chitosan (i).

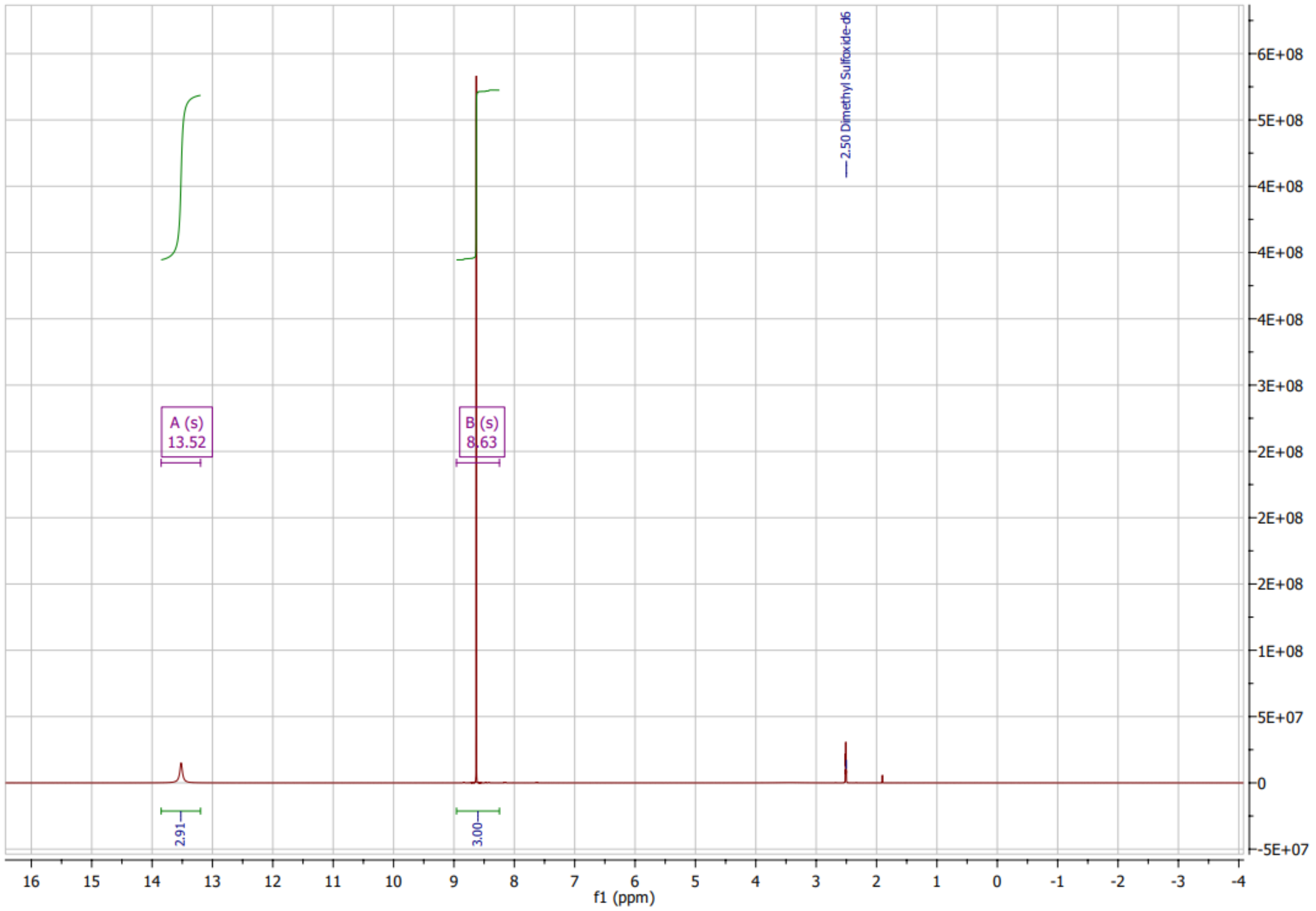

Figure S2. ^{1}H NMR spectra of BTC (iii).

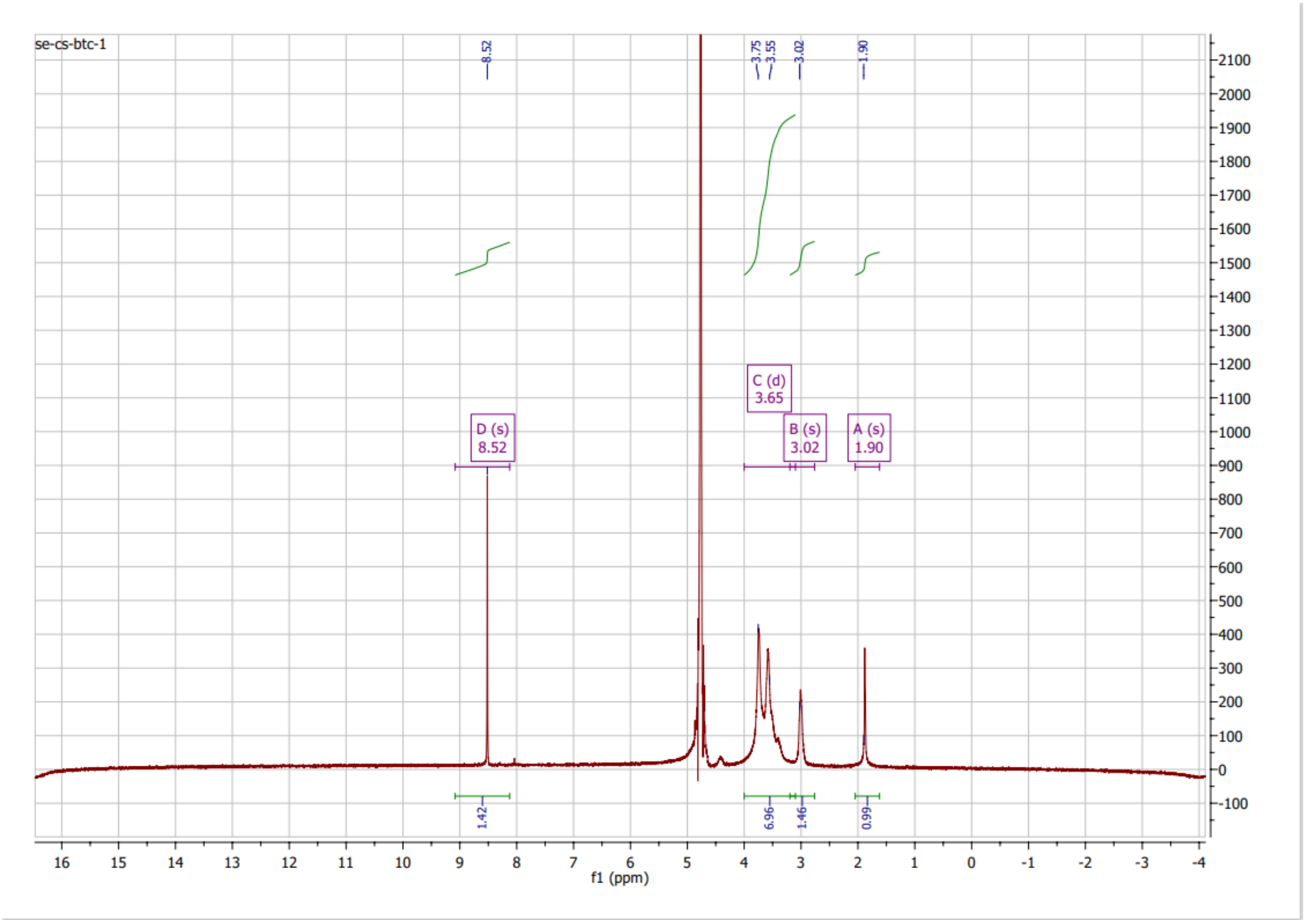

Figure S3. ^{1}H NMR spectra of chitosan glucosamine carboxylate salt (iv).

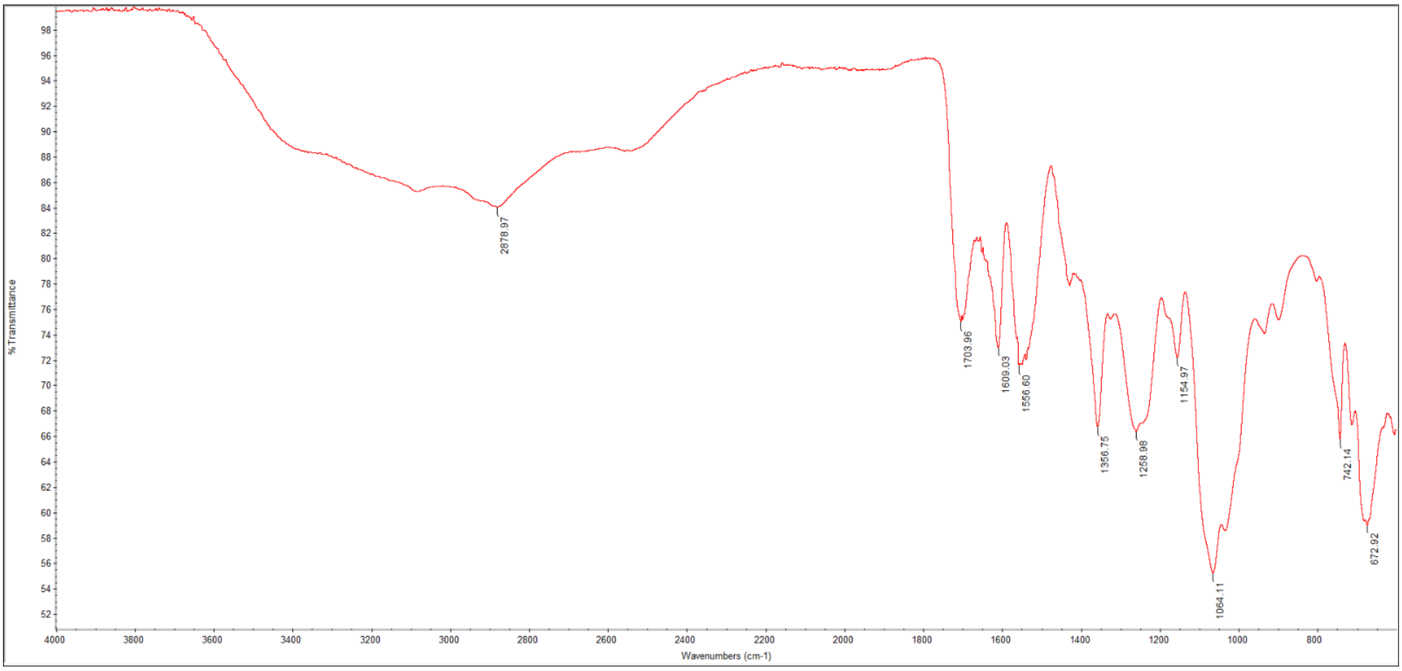

Figure S4. IR spectra of M1 hydrogel.

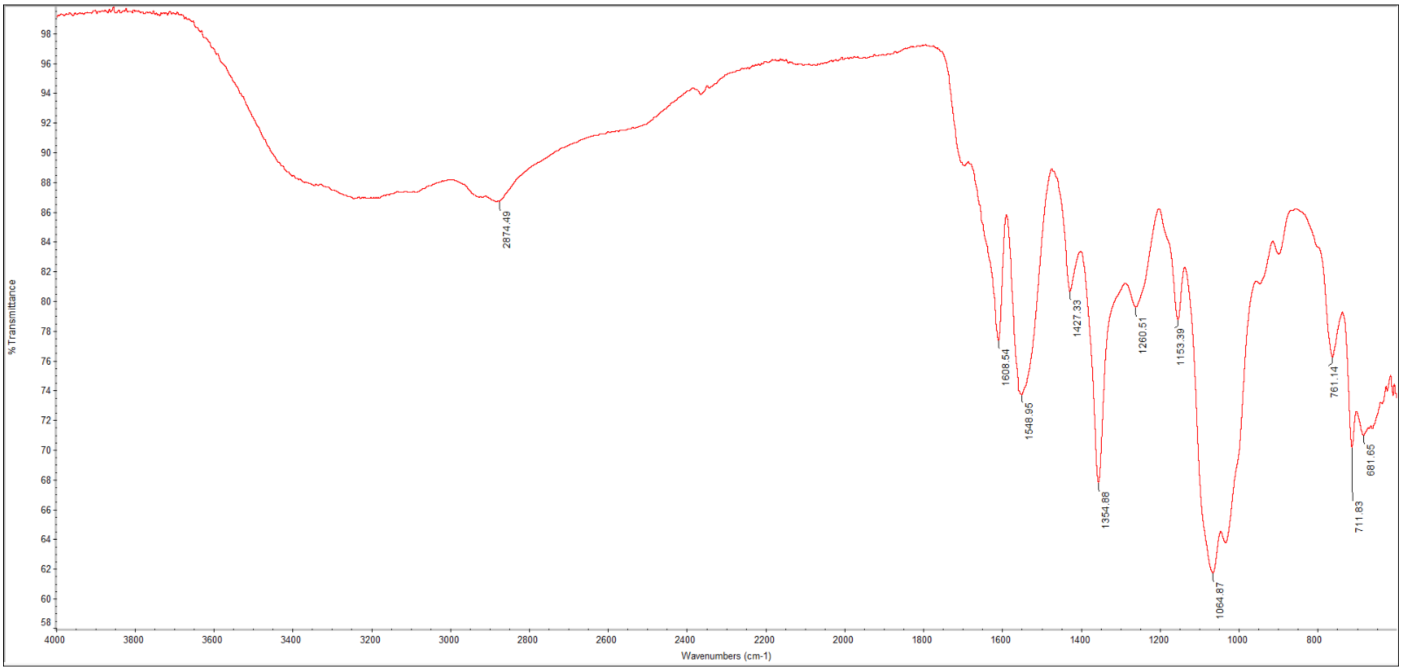

Figure S5. IR of M2 hydrogel.

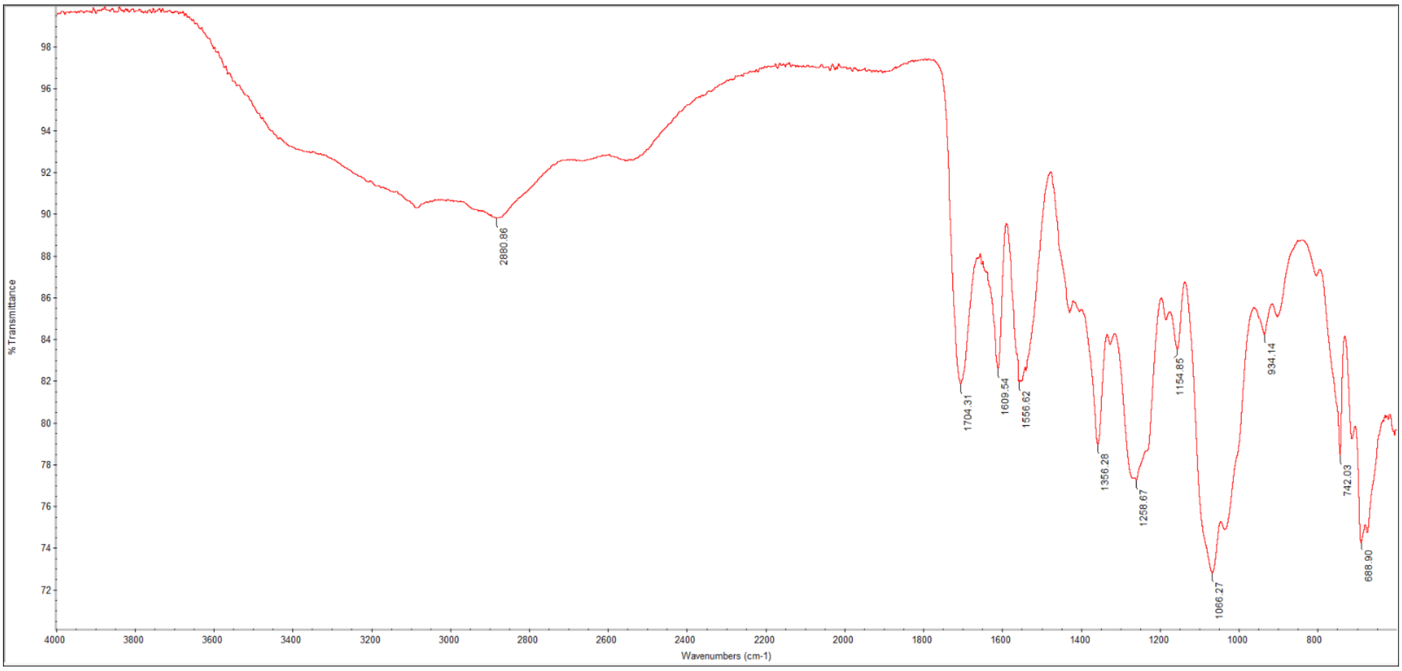

Figure S6. IR spectra of M3 hydrogel.

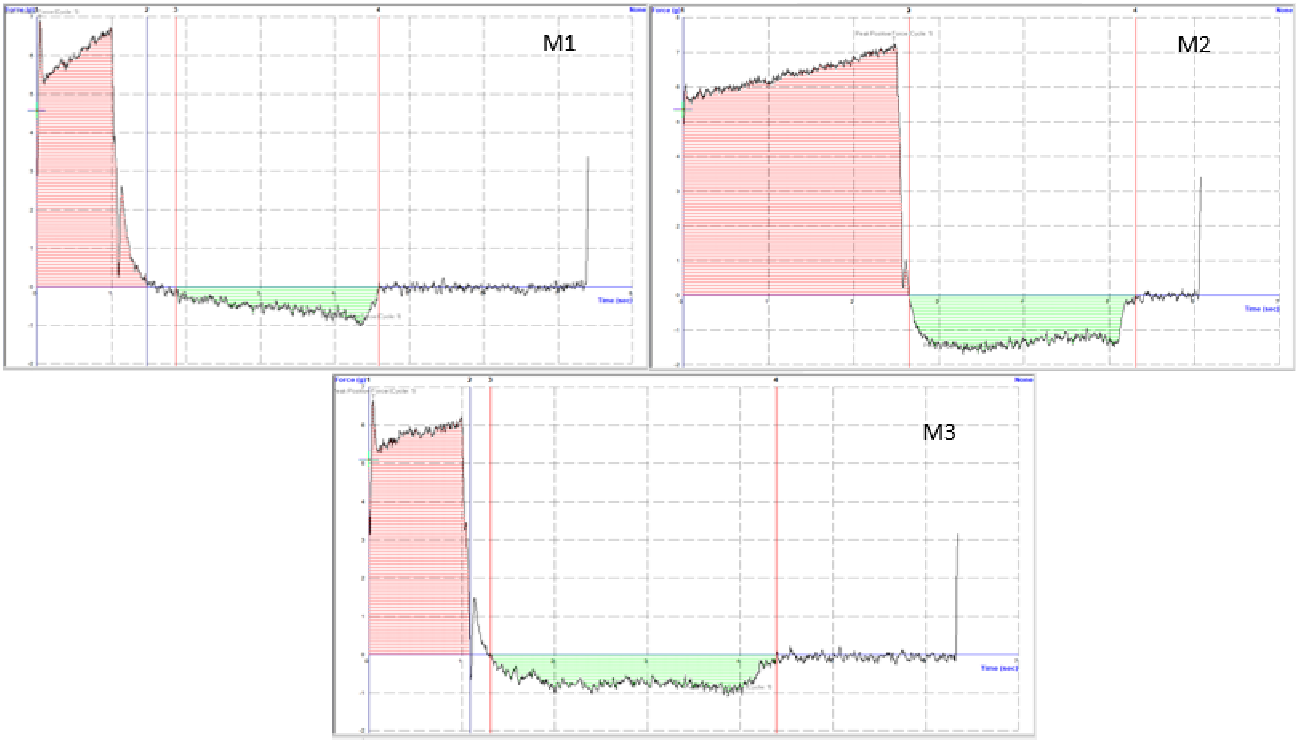

Figure S7. Force vs. time plot of chitosan–BTC hydrogels in three different concentrations: M1, M2, and M3.

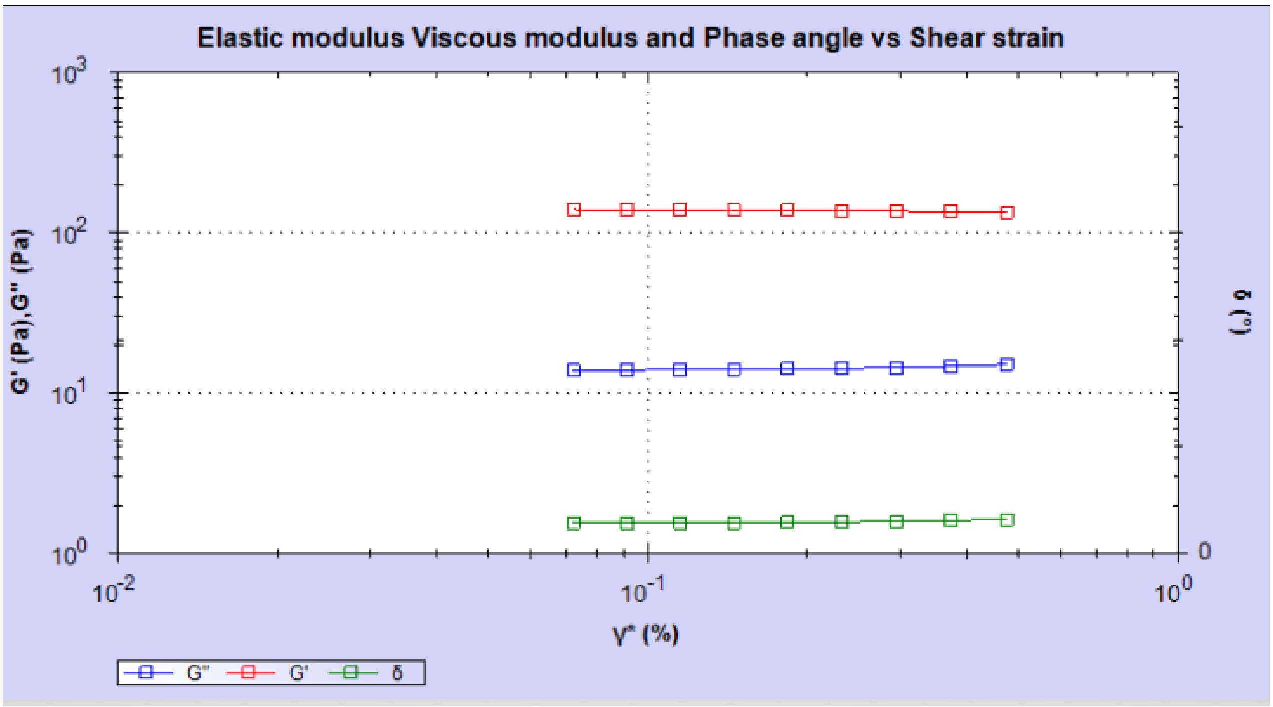

Figure S8. Oscillation dynamics of elastic and viscous moduli of chitosan cross-linked with BTC (M1 hydrogel).

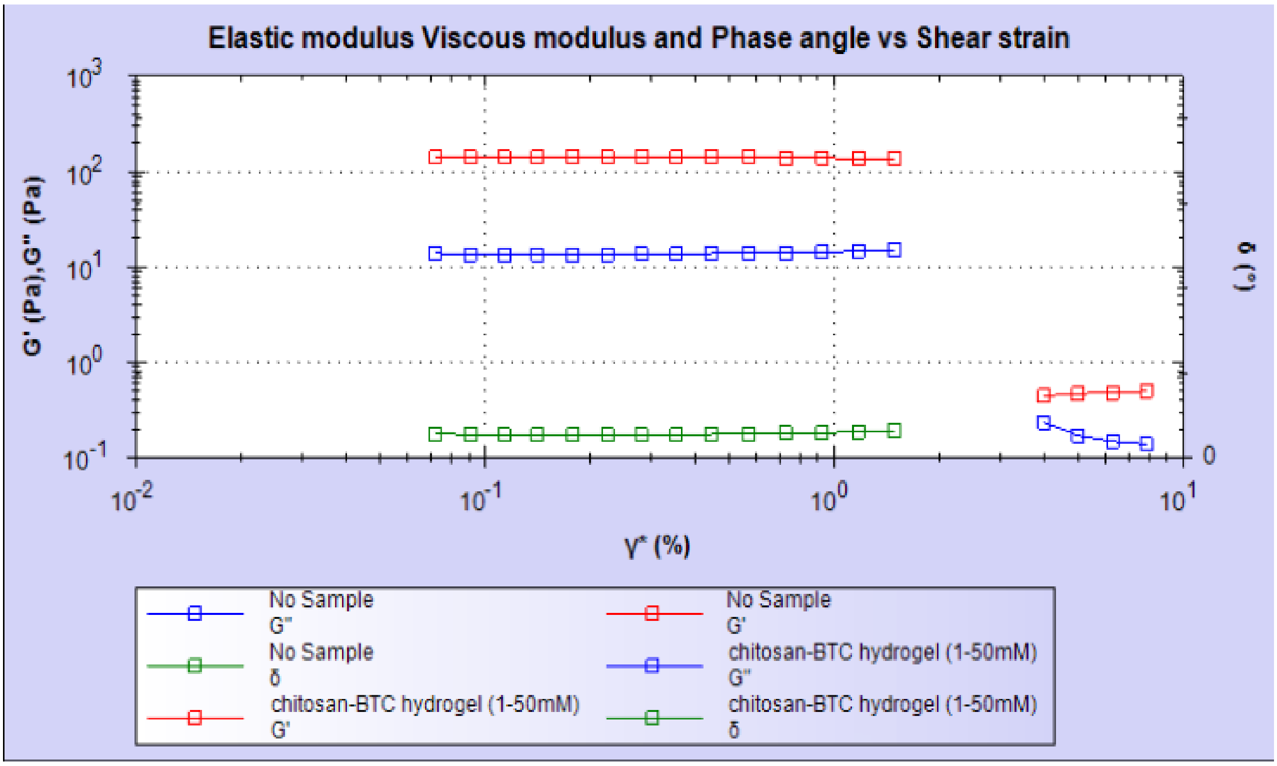

Figure S9. Oscillation dynamics of elastic and viscous moduli of chitosan cross-linked with BTC (M2 hydrogel).

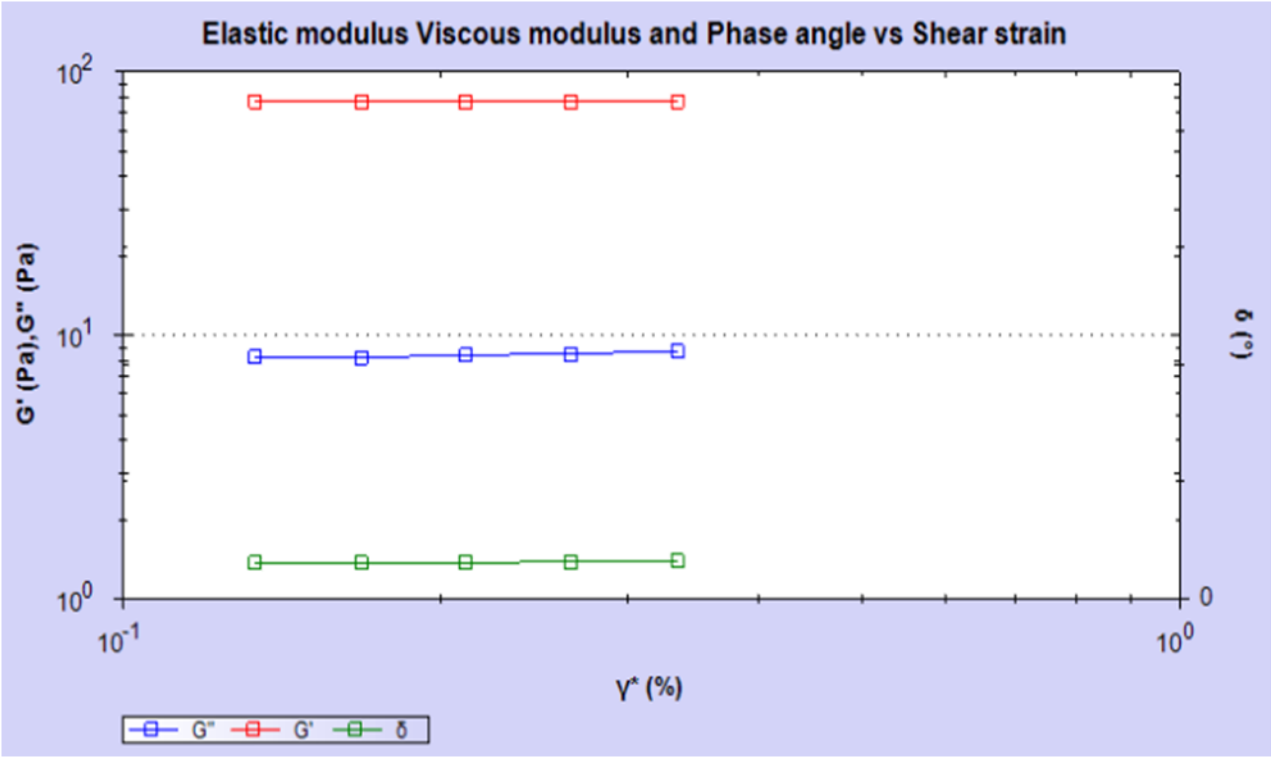

Figure S10. Oscillation dynamics of elastic and viscous moduli of chitosan cross-linked with BTC (M3 hydrogel).

Table S1. Hydrogels compositions.

Chitosan Gels	Chitosan (g)	Acetic Acid (mL)	BTC (g)	Ethanol (mL)
**M1**	2.5	250	2.625	250
**M2**	2.5	250	0.525	50
**M3**	1.25	125	2.625	250

Table S2. Amplitude strain sweep for hydrogels with chitosan–BTC concentration 1–10 Mm (supplementary material).

Complex Shear Strain (%)	Complex Shear Stress (Pa)	Shear Modulus (Complex Component) (Pa)	Shear Modulus (Elastic Component) (Pa)	Shear Modulus (Viscous Component) (Pa)	Shear Viscosity (Complex Component) (Pa s)	Phase Angle (Â°)	Time (s)	Torque (N m)
**0.072263**	0.1018	140.9	140.2	13.91	22.43	5.66	20	1.71×10^{-6}
**0.091081**	0.1282	140.8	140.1	13.95	22.4	5.69	40.01	2.15×10^{-6}
**0.114901**	0.1614	140.5	139.8	14.03	22.36	5.73	60.02	2.70×10^{-6}
**0.14512**	0.2032	140.0	139.3	14.06	22.29	5.76	80.04	3.41×10^{-6}
**0.183392**	0.2558	139.5	138.8	14.16	22.20	5.83	100.1	4.29×10^{-6}
**0.232032**	0.3221	138.8	138.1	14.25	22.09	5.89	120.1	5.40×10^{-6}
**0.293968**	0.4056	138.0	137.2	14.44	21.96	6.01	140.1	6.80×10^{-6}
**0.372866**	0.5106	136.9	136.2	14.73	21.80	6.18	160.1	8.56×10^{-6}
**0.474208**	0.643	135.6	134.7	15.11	21.58	6.40	180.1	1.08×10^{-5}

Table S3. Amplitude strain sweep for hydrogels with chitosan–BTC concentration 1–50 mM (supplementary material).

Complex Shear Strain (%)	Complex Shear Stress (Pa)	Shear Modulus (Complex Component) (Pa)	Shear Modulus (Elastic Component) (Pa)	Shear Modulus (Viscous Component (Pa)	Shear Viscosity (Complex Component) (Pa s)	Phase Angle (Â°)	Torque (N m)
**0.071935**	0.1018	141.5	140.9	13.94	22.53	5.65	1.71×10^{-6}
**0.09063**	0.1282	141.4	140.8	13.49	22.51	5.47	2.15×10^{-6}
**0.11339**	0.1614	142.3	141.7	13.32	22.65	5.37	2.7×10^{-6}
**0.141341**	0.2031	143.7	143.1	13.39	22.87	5.35	3.4×10^{-6}
**0.177932**	0.2557	143.7	143.1	13.38	22.87	5.34	4.28×10^{-6}
**0.223731**	0.3219	143.9	143.2	13.45	22.9	5.36	5.39×10^{-6}
**0.280822**	0.4052	144.3	143.7	13.58	22.97	5.40	6.79×10^{-6}
**0.35369**	0.5102	144.2	143.6	13.71	22.96	5.45	8.55×10^{-6}
**0.4465**	0.6423	143.8	143.2	13.86	22.89	5.53	1.08×10^{-5}
**0.568814**	0.8088	142.2	141.5	13.95	22.63	5.63	1.36×10^{-5}
**0.726616**	1.018	140.2	139.5	14.07	22.31	5.76	1.71×10^{-5}
**0.925929**	1.282	138.5	137.8	14.27	22.04	5.91	2.15×10^{-5}
**1.17428**	1.615	137.5	136.7	14.66	21.88	6.12	2.71×10^{-5}
**1.48867**	2.033	136.6	135.7	15.07	21.74	6.34	3.41×10^{-5}

Table S4. Amplitude strain sweep for hydrogels with chitosan–BTC concentration 0.5–50 Mm (supplementary material).

Complex Shear Strain (%)	Complex Shearstress (Pa)	Shear Modulus (Complex Component) (Pa)	Shear Modulus (Elastic Component) (Pa)	Shear Modulus (Viscous Component) (Pa)	Shear Viscosity (Complex Component) (Pa s)	Phase Angle (Â°)	Torque (N m)
**0.133373**	0.1034	77.51	77.07	8.306	12.34	6.15	1.73×10^{-6}
**0.168133**	0.1302	77.41	76.97	8.240	12.32	6.11	2.18×10^{-6}
**0.210965**	0.1638	77.66	77.2	8.411	12.36	6.22	2.75×10^{-6}
**0.265304**	0.2063	77.74	77.27	8.512	12.37	6.29	3.46×10^{-6}
**0.334426**	0.2597	77.64	77.15	8.711	12.36	6.44	4.35×10^{-6}

Table S5. Frequency sweep for hydrogels with chitosan–BTC concentration 1–10 mM (supplementary material).

Frequency (Hz)	Complex Shear Strain (%)	Complex Shear Stress (Pa)	Shear Modulus (Complex Component) (Pa)	Shear Modulus (Elastic Component) (Pa)	Shear Modulus (Viscous Component) (Pa)	Shear Viscosity (Complex Component) (Pa s)	Phase Angle (Â°)	Torque (N m)
10	0.88081	1.605	182.2	179.8	29.25	2.90	9.24	2.69×10^{-5}
7.943	0.87749	1.545	176.1	173.7	28.81	3.52	9.42	2.59×10^{-5}
6.31	0.92754	1.594	171.9	169.6	27.66	4.33	9.26	2.67×10^{-5}
5.012	0.97117	1.631	167.9	165.7	27.11	5.33	9.29	2.73×10^{-5}
3.981	0.97312	1.600	164.4	162.3	26.37	6.57	9.23	2.68×10^{-5}
3.162	0.99236	1.600	161.2	159.1	25.81	8.11	9.21	2.68×10^{-5}
2.512	1.00053	1.579	157.8	155.8	25.15	9.99	9.17	2.65×10^{-5}
1.995	1.00293	1.549	154.5	152.5	24.41	12.32	9.09	2.60×10^{-5}
1.585	1.00225	1.519	151.6	149.7	23.92	15.22	9.08	2.55×10^{-5}
1.259	1.00308	1.490	148.5	146.6	23.46	18.77	9.09	2.50×10^{-5}
1	1.00023	1.458	145.8	143.9	23.14	23.20	9.13	2.44×10^{-5}
0.7943	1.00047	1.431	143.1	141.2	22.77	28.67	9.16	2.40×10^{-5}
0.631	1.00238	1.407	140.4	138.6	22.41	35.40	9.19	2.36×10^{-5}
0.5012	0.99951	1.378	137.9	136.1	22.14	43.78	9.24	2.31×10^{-5}
0.3981	1.00082	1.356	135.4	133.7	21.87	54.15	9.29	2.27×10^{-5}
0.3162	1.00148	1.334	133.2	131.4	21.50	67.02	9.29	2.23×10^{-5}
0.2512	1.00089	1.311	131	129.3	21.16	83.01	9.29	2.20×10^{-5}
0.1995	1.00018	1.291	129.1	127.4	20.86	103	9.3	2.16×10^{-5}
0.1585	1.00076	1.273	127.2	125.6	20.59	127.8	9.31	2.13×10^{-5}

Table S6. Frequency strain sweep for hydrogels with chitosan–BTC concentration 1–50 mM (supplementary material).

Frequency (Hz)	Complex shear strain (%)	Complex shear stress (Pa)	Shear modulus (complex component) (Pa)	Shear modulus (elastic component) (Pa)	Shear modulus (viscous component) (Pa)	Shear viscosity (complex component) (Pa s)	Phase angle (Â°)	Torque (N m)
**10**	0.925802	1.123	121.3	119.9	18.18	1.93	8.62	1.88E-05
**7.943**	0.948839	1.111	117.1	115.7	17.91	2.346	8.8	1.86E-05
**6.31**	0.927143	1.063	114.6	113.4	16.64	2.891	8.35	1.78E-05
**5.012**	0.96588	1.083	112.1	111	15.99	3.561	8.2	1.82E-05
**3.981**	0.972022	1.068	109.9	108.8	15.35	4.393	8.03	1.79E-05
**3.162**	0.991419	1.07	108	107	14.68	5.433	7.82	1.79E-05
**2.512**	0.999767	1.059	105.9	105	14.21	6.712	7.71	1.78E-05
**1.995**	1.0027	1.041	103.9	103	13.61	8.285	7.53	1.75E-05
**1.585**	1.0019	1.024	102.2	101.3	13.18	10.26	7.41	1.72E-05
**1.259**	1.00231	1.007	100.4	99.63	12.77	12.7	7.3	1.69E-05
**1**	1.00048	0.9902	98.98	98.19	12.49	15.75	7.25	1.66E-05
**0.7943**	1.00114	0.9755	97.44	96.68	12.14	19.52	7.16	1.63E-05
**0.631**	1.00153	0.9597	95.82	95.09	11.84	24.17	7.09	1.61E-05
**0.5012**	0.999925	0.9449	94.5	93.78	11.63	30.01	7.07	1.58E-05
**0.3981**	1.00077	0.932	93.13	92.44	11.33	37.23	6.99	1.56E-05
**0.3162**	1.00124	0.9195	91.83	91.16	11.12	46.22	6.95	1.54E-05
**0.2512**	1.00099	0.906	90.51	89.83	11	57.35	6.98	1.52E-05
**0.1995**	0.999752	0.8931	89.33	88.68	10.79	71.26	6.93	1.50E-05
**0.1585**	1.00028	0.8831	88.28	87.64	10.64	88.65	6.92	1.48E-05
**0.1259**	1.00032	0.8721	87.18	86.54	10.53	110.2	6.94	1.46E-05
**0.1**	0.997762	0.8596	86.15	85.53	10.38	137.1	6.92	1.44E-05

Table S7. Frequency strain sweep for hydrogels with chitosan–BTC concentration 0.5–50 mM (supplementary material).

Frequency (Hz)	Complex Shear Strain (%)	Complex Shear Stress (Pa)	Shear Modulus (Complex Component) (Pa)	Shear Modulus (Elastic Component) (Pa)	Shear Modulus (Viscous Component) (Pa)	Shear Viscosity (Complex Component) (Pa s)	Phase Angle (Â°)	Torque (N m)
**10**	0.91961	0.7309	79.48	78.12	14.65	1.265	10.62	1.23×10^{-5}
**7.943**	0.97892	0.7517	76.79	75.54	13.77	1.539	10.33	1.26×10^{-5}
**6.31**	0.95323	0.718	75.32	74.22	12.85	1.9	9.82	1.20×10^{-5}
**5.012**	0.96221	0.7092	73.71	72.66	12.38	2.341	9.67	1.19×10^{-5}
**3.981**	0.97024	0.699	72.04	71.06	11.85	2.88	9.47	1.17×10^{-5}
**3.162**	0.99083	0.6994	70.59	69.65	11.45	3.553	9.34	1.17×10^{-5}
**2.512**	1.00014	0.6909	69.08	68.2	11.01	4.377	9.17	1.16×10^{-5}
**1.995**	1.00287	0.6784	67.64	66.81	10.6	5.396	9.02	1.14×10^{-5}

**1.585**	1.00168	0.6642	66.31	65.51	10.28	6.659	8.91	1.11×10^{-5}
**1.259**	1.0028	0.652	65.02	64.25	9.933	8.22	8.79	1.09×10^{-5}
**1**	1.00032	0.6387	63.85	63.12	9.646	10.16	8.69	1.07×10^{-5}
**0.7943**	1.00098	0.6275	62.69	61.98	9.382	12.56	8.61	1.05×10^{-5}
**0.631**	1.00217	0.6171	61.57	60.9	9.1	15.53	8.5	1.03×10^{-5}
**0.5012**	1.00038	0.6056	60.54	59.88	8.877	19.22	8.43	1.02×10^{-5}
**0.3981**	1.00075	0.596	59.55	58.92	8.644	23.81	8.35	9.99×10^{-6}
**0.3162**	1.00069	0.5867	58.63	58.02	8.424	29.51	8.26	9.83×10^{-6}
**0.2512**	1.00114	0.5784	57.77	57.19	8.204	36.6	8.16	9.69×10^{-6}
**0.1995**	1.00065	0.5704	57.01	56.45	7.979	45.47	8.05	9.56×10^{-6}
**0.1585**	1.00056	0.5633	56.29	55.75	7.801	56.53	7.97	9.44×10^{-6}
**0.1259**	1.00153	0.5569	55.6	55.08	7.572	70.29	7.83	9.33×10^{-6}
**0.1**	0.99938	0.5496	55	54.49	7.449	87.53	7.78	9.21×10^{-6}

Table S8. Frequency strain sweep for hydrogels with chitosan–BTC concentration 0.5–50 mM (supplementary material).

Frequency (Hz)	Complex Shear Strain (%)	Complex Shear Stress (Pa)	Shear Modulus (Complex Component) (Pa)	Shear Modulus (Elastic Component) (Pa)	Shear Modulus (Viscous Component) (Pa)	Shear Viscosity (Complex Component) (Pa s)	Phase Angle (Â°)	Torque (N m)
**10**	0.919613	0.7309	79.48	78.12	14.65	1.265	10.62	1.23×10^{-5}
**7.943**	0.978926	0.7517	76.79	75.54	13.77	1.539	10.33	1.26×10^{-5}
**6.31**	0.953236	0.718	75.32	74.22	12.85	1.9	9.82	1.20×10^{-5}
**5.012**	0.962218	0.7092	73.71	72.66	12.38	2.341	9.67	1.19×10^{-5}
**3.981**	0.970245	0.699	72.04	71.06	11.85	2.88	9.47	1.17×10^{-5}
**3.162**	0.990843	0.6994	70.59	69.65	11.45	3.553	9.34	1.17×10^{-5}
**2.512**	1.00014	0.6909	69.08	68.2	11.01	4.377	9.17	1.16×10^{-5}
**1.995**	1.00287	0.6784	67.64	66.81	10.6	5.396	9.02	1.14×10^{-5}
**1.585**	1.00168	0.6642	66.31	65.51	10.28	6.659	8.91	1.11×10^{-5}
**1.259**	1.0028	0.652	65.02	64.25	9.933	8.22	8.79	1.09×10^{-5}
**1**	1.00032	0.6387	63.85	63.12	9.646	10.16	8.69	1.07×10^{-5}
**0.7943**	1.00098	0.6275	62.69	61.98	9.382	12.56	8.61	1.05×10^{-5}
**0.631**	1.00217	0.6171	61.57	60.9	9.1	15.53	8.5	1.03×10^{-5}
**0.5012**	1.00038	0.6056	60.54	59.88	8.877	19.22	8.43	1.02×10^{-5}
**0.3981**	1.00075	0.596	59.55	58.92	8.644	23.81	8.35	9.99×10^{-6}
**0.3162**	1.00069	0.5867	58.63	58.02	8.424	29.51	8.26	9.83×10^{-6}
**0.2512**	1.00114	0.5784	57.77	57.19	8.204	36.6	8.16	9.69×10^{-6}
**0.1995**	1.00065	0.5704	57.01	56.45	7.979	45.47	8.05	9.56×10^{-6}
**0.1585**	1.00056	0.5633	56.29	55.75	7.801	56.53	7.97	9.44×10^{-6}
**0.1259**	1.00153	0.5569	55.6	55.08	7.572	70.29	7.83	9.33×10^{-6}
**0.1**	0.999381	0.5496	55	54.49	7.449	87.53	7.78	9.21×10^{-6}
